# Identification of a Dolabellane Type Diterpene Synthase and other Root-Expressed Diterpene Synthases in *Arabidopsis*

**DOI:** 10.3389/fpls.2016.01761

**Published:** 2016-11-25

**Authors:** Qiang Wang, Meirong Jia, Jung-Hyun Huh, Andrew Muchlinski, Reuben J. Peters, Dorothea Tholl

**Affiliations:** ^1^Department of Biological Sciences, Virginia Tech, BlacksburgVA, USA; ^2^Roy J. Carver Department of Biochemistry, Biophysics and Molecular Biology, Iowa State University, AmesIA, USA

**Keywords:** *Arabidopsis*, diterpenes, ecotype, root, terpene synthase

## Abstract

*Arabidopsis thaliana* maintains a complex metabolism for the production of secondary or specialized metabolites. Such metabolites include volatile and semivolatile terpenes, which have been associated with direct and indirect defensive activities in flowers and leaves. In comparison, the structural diversity and function of terpenes in *Arabidopsis* roots has remained largely unexplored despite a substantial number of root-expressed genes in the *Arabidopsis* terpene synthase (*TPS*) gene family. We show that five root-expressed *TPSs* of an expanded subfamily-a type clade in the *Arabidopsis TPS* family function as class I diterpene synthases that predominantly convert geranylgeranyl diphosphate (GGPP) to different semi-volatile diterpene products, which are in part detectable at low levels in the ecotypes Columbia (Col) and Cape Verde Island (Cvi). The enzyme TPS20 produces a macrocyclic dolabellane diterpene alcohol and a dolabellane-related diterpene olefin named dolathaliatriene with a so far unknown C6-C11 bicyclic scaffold besides several minor olefin products. The TPS20 compounds occur in all tissues of Cvi but are absent in the Col ecotype because of deletion and substitution mutations in the Col TPS20 sequence. The primary TPS20 diterpene products retard the growth of the root rot pathogen *Pythium irregulare* but only at concentrations exceeding those *in planta*. Together, our results demonstrate that divergence and pseudogenization in the *Arabidopsis TPS* gene family allow for structural plasticity in diterpene profiles of above- and belowground tissues.

## Introduction

Among the many specialized metabolites that are synthesized by plants, terpenes exhibit some of the highest biosynthetic and structural diversity (Degenhardt et al., 2009; Thimmappa et al., 2014; Zi et al., 2014; Lange, 2015; Tholl, 2015). This diversity is reflective of the multiple biological roles of terpene compounds in the attraction of pollinators (Byers et al., 2014), in direct and indirect defense against herbivores and pathogens (Kessler and Baldwin, 2001; Unsicker et al., 2009; Hall et al., 2011; Schmelz et al., 2011; Huang et al., 2012; Mithöfer and Boland, 2012; Quintana-Rodriguez et al., 2015), as signals in systemic acquired resistance or inter/intra-plant communication (Arimura et al., 2000; Heil and Silva Bueno, 2007; Karban and Shiojiri, 2009; Chaturvedi et al., 2012), and in the protection against abiotic stress (Loreto et al., 2001; Ryan et al., 2014; Vaughan et al., 2015). To facilitate such interactions at short and long distance, plants often employ volatile or semi-volatile terpenes of low molecular weight that include the 5-carbon hemiterpenes, 10-carbon monoterpenes, 15-carbon sesquiterpenes, and 20-carbon diterpenes (Dudareva et al., 2006). These compounds are produced by large families of terpene synthases (TPSs) from the central terpene biosynthetic precursors dimethylallyl diphosphate (DMAPP, C5), geranyl or neryl diphosphate (GPP, NPP, C10), *cis* or *trans* farnesyl diphosphate (FPP, C15), and geranylgeranyl or copalyl diphosphate (GGPP, CPP, C20), respectively (Chen et al., 2011). The biosynthesis and function of volatile terpenes have been investigated primarily in aboveground plant tissues (e.g., Schnee et al., 2006; Danner et al., 2011; Zhuang et al., 2012; Byers et al., 2014). However, comparatively few studies have shown to what extent the diversity of volatile or semi-volatile terpene metabolism and function in plant roots resembles that of leaves and flowers or varies depending on the exposure of plant tissues to different environments above and belowground (Köllner et al., 2008; Chen et al., 2009).

*Arabidopsis thaliana* maintains a rather complex terpene specialized metabolism that includes the constitutive or stress-induced production of an array of volatile and semi-volatile terpene compounds in flowers (Chen et al., 2003; Tholl et al., 2005; Ginglinger et al., 2013), leaves (Aharoni et al., 2003; Huang et al., 2010; Lee et al., 2010; Snoeren et al., 2010), and roots (Steeghs et al., 2004; Vaughan et al., 2013; Sohrabi et al., 2015). In aboveground tissues, volatile terpenes and their non-volatile derivatives have been implicated in defensive activities. For example, flowers produce the volatile sesquiterpene (*E*)-β-caryophyllene and oxygenated derivatives of the monoterpene alcohol, linalool, that aid in protecting reproductive tissues against attack by microbial pathogens or small herbivores (Huang et al., 2012; Boachon et al., 2015). *Arabidopsis* leaves emit monoterpenes, sesquiterpenes, and the irregular homoterpene, TMTT (*E,E*)-4,8,12-trimethyltrideca-1,3,7,11-tetraene) upon herbivory or pathogen invasion in a response that is likely involved in direct and indirect defense (Herde et al., 2008; Huang et al., 2010).

By contrast, much less is known about the biosynthesis of terpenes and their activities in *Arabidopsis* roots, in part because of the analytical challenges that are associated with the detection of these compounds at low concentrations in the root tissue. Within the *Arabidopsis TPS* gene family (Aubourg et al., 2002; Tholl and Lee, 2011) more than one third out of 32 genes are expressed in roots, which suggests an active terpenoid specialized metabolism in this tissue. Interestingly, 12 of the 14 root expressed genes belong to an expanded subfamily-a type clade of 22 *TPS* genes. The other root-expressed TPSs, the 1,8-cineole synthases TPS24 (At3g25810) and TPS27 (At3g25820), belong to a TPS subfamily-b type clade together with four other monoterpene synthases, which are expressed constitutively or upon biotic stress in flowers and leaves (Chen et al., 2003, 2004; Huang et al., 2010). TPS-a subfamilies of different angiosperms have been shown to contain sesquiterpene synthases (sesquiTPSs) and expanded clusters of diterpene synthases (diTPSs) (Facchini and Chappell, 1992; Mau and West, 1994; Kirby et al., 2010; Zerbe et al., 2013). Among the root-expressed TPSs of the *Arabidopsis* type-a clade two sesquiTPSs have previously been characterized as γ-bisabolene synthases (TPS12, At4g13280; TPS13, At4g13300) (Ro et al., 2006). The remaining root-expressed enzymes in this clade were predicted to be diTPSs based on their presumed targeting to plastids as the predominant sites of diterpene biosynthesis (Lichtenthaler, 2010). To date, a single root-expressed diTPS (TPS8, At4g20210) of the *Arabidopsis* TPS-a type clade has been identified, which produces the semi-volatile diterpenes, rhizathalenes, with an unusual tricyclic spiro-hydrindane structure. Rhizathalenes are released from the root stele and function as local insect feeding deterrents (Vaughan et al., 2013).

Here we describe the biochemical function of seven additional TPSs of the type-a clade with partial or predominant expression in *Arabidopsis* roots. Among these enzymes, TPS20 (At5g48110) produces semi-volatile dolabellane type and related diterpenes, which have been characterized mainly in marine organisms for their antibacterial activity (Ioannou et al., 2011). TPS20 is functionally active in the ecotype Cape Verde Island (Cvi) but is inactive in the Columbia (Col) ecotype as a consequence of deletion and substitution mutations in the Col TPS20 sequence. The Cvi TPS20 protein is targeted to the plastid and expressed in roots and aboveground tissues where its enzymatic products can be detected at low levels. TPS20 products retard the growth of the root rot pathogen, *Pythium irregulare, in vitro* in a dose dependent manner but at concentrations above those determined *in vivo*. In addition to TPS20, we show that the recombinant proteins of TPS6 (At1g70080), TPS9 (At4g20230), TPS22 (At1g33750), TPS25 (At3g29410), TPS26 (At1g66020), and TPS30 (At3g32030) from the Col or Cvi ecotypes can function as sesquiTPSs and/or diTPSs.

## Materials and Methods

### Plant Material and Treatment

*Arabidopsis thaliana*, ecotype Columbia (Col-0) and Cvi, were grown in Sunshine mix #1 (Sun Gro Horticulture) under long day condition with 14-h-light/10-h-dark photoperiod at 22–25°C. *Nicotiana benthamiana* was germinated and grown in potting substrate at 22°C with a 16 h day/8 h night photoperiod for 4–5 weeks prior to agroinfiltration. Roots of Cvi grown under axenic culture conditions (Sohrabi et al., 2015) were treated for 24 h with jasmonic acid (100 μM).

### Cloning of *TPS20* and Other *TPS* Genes

To clone the cDNA of *TPS20* (At5g48110) from the Col ecotype, RNA was extracted from roots using TRIzol (Invitrogen) following the manufacture’s protocol. cDNA was synthesized using the M-MLV reverse transcriptase kit (Promega). The full length open reading frame (ORF) of *TPS20* (At5g48110) was amplified using primers designed based on the gene sequence from TAIR^1^ (Supplementary Table S1). The cDNA of *TPS20* from Cvi (designated *TPS20c*), was cloned accordingly from RNA of Cvi flowers. The *TPS20* and *TPS20c* cDNA fragments were ligated into the pGEM-T easy vector (Promega) for sequencing. To obtain functionally fully active proteins, the first 53 amino acids of a predicted plastidial transit peptide of the TPS20 and TPS20c ORFs were removed by amplifying truncated versions of the cDNAs of both genes from the pGEM-T easy clones. The truncated cDNAs were then sub-cloned into the pET28a protein expression vector using *Nco*I and *Xho*I restriction sites. Protein expression constructs of *TPS6* (At1g70080, Cvi), *TPS19* (At3g14540), *TPS26* (At1g66020), *TPS29* (At1g31950), and *TPS30* (At3g32030) (all Col) were prepared in pET28a as described for *TPS20* and *TPS20c*, while the cDNA of *TPS9* (At4g20230) was cloned in the pET Duet vector (Novagen) and the cDNAs of *TPS22* (At1g33750) and *TPS25* (At3g29410) (both from Col) were cloned into TOPO-pET102 (Invitrogen) following the manufacturers protocols. Original cDNA clones for *TPS9* and *TPS26* were obtained from the RIKEN *Arabidopsis* full length clone collection^2^. N-terminal truncations were made for TPS6 (from Cvi, 53 amino acids), TPS19 (48 amino acids), TPS22 (41 amino acids), TPS25 (25 amino acids), TPS26 (38 amino acids), TPS29 (50 amino acids), and TPS30 (48 amino acids) to test for or obtain functionally fully active recombinant proteins. To generate a TPS20c protein with an N-terminal histidine tag fusion, the truncated TPS20c cDNA was cloned into pET28a using *NdeI* and *XhoI* restriction sites. PCR primers used for the amplification of all *TPS* cDNAs are listed in Supplementary Table S1.

### Recombinant Expression and Terpene Product Analysis

To test the potential diTPS and/or sesquiTPS activities of TPS20 and TPS20c in bacterial culture, the truncated cDNAs of both genes cloned in pET28a were heterologously expressed in *Escherichia coli* BL21 together with a GGPP synthase from *Abies grandis* (grand fir) (in a pGG construct), a GGPP and *ent*-CPP synthase (in a pGGeC construct), or a (*E,E*)-FPP synthase from *E. coli* (in a pACYC-Duet/IspA construct) using a previously described expression system for diterpene production (Fujisaki et al., 1990; Cyr et al., 2007). Terpene products were obtained by extracting 50 mL bacterial culture twice with equal volumes of hexane, and the pooled organic solvent was subsequently concentrated by rotary evaporation. The residues were re-suspended in 0.5 mL hexane and 1 μL was analyzed by GC-MS. Terpene product analysis was performed with a GC-2010 gas chromatograph coupled with a quadrupole mass spectrometer (GC-MS-QP2010S; Shimadzu) in splitless mode as described before (Vaughan et al., 2013; Wang et al., 2014). Compounds were separated with a temperature gradient of 40°C for 2 min, 5°C/min gradient to 240°C, and 2 min at 240°C. Recombinant proteins of the other *Arabidopsis* TPSs except TPS22 and TPS25 were expressed in *E. coli* and characterized with the same procedure. For a comparison of the terpene products of TPS6 and TPS20 and the analysis of TPS30 products (performed by the Peters group) a Varian 3900 GC with a Saturn 2100T ion trap mass spectrometer (injection port 250°C) and Agilent HP-5MS column (1.2 mL/min helium flow rate) was used and a temperature program of 50°C (3 min), 15°C/min to 300°C (hold 3 min) was applied. To obtain distinct product profiles for TPS22 and TPS25, their recombinant proteins were expressed and partially purified following previously described protocols (Tholl et al., 2005; Huang et al., 2010). Analysis of the TPS22 and TPS25 enzymatic products was performed with SPME-GC-MS as described (Tholl et al., 2005; Huang et al., 2010) Where possible, products of all the expressed TPS proteins were identified by comparison of GC retention times and mass spectra to those of authentic standards and mass spectra of the Wiley/NIST libraries.

### TPS20c Diterpene Production and Purification

To obtain sufficient enzymatic product from TPS20c for NMR analysis, *E. coli* C41 OverExpress (Lucigen) was co-transformed with the truncated TPS20c, pGG and the pIRS plasmid, which increases isoprenoid precursor supply by overexpressing the corresponding enzymes, leading to an increased flux in terpene formation (Cyr et al., 2007; Morrone et al., 2010). The recombinant bacteria were grown to OD_600_ ∼0.8 at 37°C in 1L Terrific Broth medium (with the appropriate antibiotics) and then transferred to 16°C for 0.5 h prior to induction with IPTG (1 mM) and supplementation with pyruvate (50 mM) and MgCl_2_ (1 mM). After growing for 72 h at 16°C, the culture was extracted twice with an equal volume of hexane. The pooled organic phase was dried by rotary evaporation and the residue was re-suspended in 5 mL hexane and subsequently fractionated by flash chromatography over a 4 g-silica column using a Reveleris system (Grace, Deerfield, IL, USA) at a 15 mL/min flow rate. After sample loading, the column was washed with 100% hexane (0–4 min). The percentage of acetone was then increased to 100% (4–5 min), followed by a 100% acetone wash (5–8 min) with peak based fraction collection (15 mL maximum). Fractions of interest containing diterpene hydrocarbon and alcohol products as determined by GC-MS analysis, were dried under N_2_, and the compounds were purified by HPLC using an Agilent 1200 series instrument equipped with a Kromasil^®^ C8 HPLC column (50 mm × 4.6 mm, 5 μm) and a diode array UV detector at a flow rate of 0.5 mL/min. After sample loading, the column was washed with 50% acetonitrile/water for the diterpene alcohol product or 80% acetonitrile/water for the diterpene hydrocarbon products (0–2 min); then, the percentage of acetonitrile was increased to 100% (2–10 min) followed by a 100% acetonitrile wash (10–30 min) with collection of 0.5 mL fractions. Fractions containing pure products, as identified by GC-MS analysis, were dried under N_2_, and then dissolved in 0.5 mL benzene-*d*6 (Sigma–Aldrich) for NMR analysis.

### Chemical Structure Identification of the TPS20c Products

For structural identification of the TPS20c diterpene alcohol product, a Bruker AVII-700 spectrometer equipped with a 5-mm HCN cryogenic probe was used to record NMR spectra. The sample was analyzed at 25°C in a Shigemi NMR microtube, and chemical shifts were calculated by reference to those known for benzene-*d*_6_ [^13^C 128.39 ppm, ^1^H 7.16 ppm] signals offset from TMS. 1D ^1^H-NMR, and 2D DQF-COSY, HMQC-COSY, HSQC, HMBC and NOESY spectra were acquired at 700 MHz, while 1D ^13^C-NMR and DEPT 135 data were acquired at 174 MHz, using standard analytical parameters from the Bruker TopSpin 2.1 software. Observed HMBC correlations were used to propose a partial structure, while COSY correlations between protonated carbons were used to complete the structure, which was further verified by HSQC and DEPT 135 spectra. Correlations from observed NOESY dipole-dipole signals were used to assign stereochemistry and double bond configuration. For the diterpene hydrocarbon product, the spectra were recorded on a Bruker AVIII-800 spectrometer equipped with a 5-mm HCN cryogenic probe and installed TopSpin 3.2 software. Otherwise, the same analytical procedures were used as with the diterpene alcohol product. Assignment of the diterpene alcohol compound as (3*E*,7*E*)-dolabella-3,7-dien-18-ol was confirmed by comparison to previously reported chemical shift data (Cai et al., 2010), which are consistent with those collected here.

### Organic Extraction and Qualitative Analysis of Diterpenes from Plant Tissues

For terpene extraction from Cvi plant tissue, an entire 4-week-old *Arabidopsis* plant with flowers and cleaned roots (around 2 g) was ground in liquid nitrogen to a fine powder. The ground tissue was extracted twice under stirring with a 50 mL ethyl acetate/hexane mixture (1:1). The extract was then concentrated under rotary evaporation, re-suspended in 5 mL hexane, and loaded on a silica gel chromatography column for partial purification. The target diterpene compounds were eluted using a 10 mL hexane/ethyl acetate mixture (10:1) and concentrated to 200 μL under a gentle stream of nitrogen for subsequent GC-MS analysis. The GC temperature program was modified as follows to shorten the analysis time as described before (Wang et al., 2014): 70°C for 2 min, 10°C/min gradient to 300°C, 2 min hold at 300°C. In addition to the extraction of terpenes from whole Cvi plants, emissions of the semi-volatiles diterpene compounds from flower, leaf, and root tissues were determined using the static SPME-GC/MS analytical procedure as described by Vaughan et al. (2013). To extract and analyze terpenes from Col root tissue, the same procedures were applied as described for Cvi.

### Subcellular Localization of the TPS20c Protein

To determine the subcellular localization of the TPS20c protein, a C-terminal eGFP fusion protein was transiently expressed in leaves of *N. benthamiana*. The N-terminal transit peptide (159 bp) and the full length coding region of *TPS20c* without the stop codon were each subcloned into the pENTR/D-TOPO vector (Invitrogen) and recombined into the binary vector pK7FWG2 carrying a 35S CaMV promoter. The constructs were transformed into *Agrobacterium tumefaciens* GV3101 and infiltrated into tobacco leaves as described before (Fu et al., 2016). To prevent post-translational degradation of the eGFP fusion protein, P19 was co-infiltrated (Le Mauff et al., 2016). After 3 days, the transformed leaves were detached for fluorescence analysis using a LSM510 confocal laser scanning microscope (Carl Zeiss) as described before (Vaughan et al., 2013).

### Gene Expression Analysis

Five-week-old *Arabidopsis* Cvi plants were harvested and separated into root, leaf and flower tissues. Cvi roots treated with jasmonate in axenic culture were harvested after 24 h of hormone application. All tissues of Cvi were used for RNA extraction and cDNA synthesis as described above. RT-PCR analysis was carried out to investigate *TPS* gene expression in Cvi tissues using the primers listed in Supplementary Table S1. *Actin 8* was used as endogenous control.

### TPS20c Enzyme Kinetic Analysis

Enzyme assays were conducted with the truncated recombinant TPS20c protein carrying an N-terminal His-tag as described previously (Tholl et al., 2005). Assays were performed in a final volume of 1 mL with 1 μg partially purified TPS20c enzyme and 10 μM [1-^3^H]-GGPP (0.74 TBq mmol^-1^). Assay conditions and quantification of the radioactive products were as described before (Tholl et al., 2005). Six different concentrations of [1-^3^H]-GGPP were applied to determine the *K*_m_ value for GGPP in triplicate assays. Calculation of *K*_m_ and *V*_max_ values was performed as described before (Tholl et al., 2005; Vaughan et al., 2013).

### Growth Inhibition Assays with *Pythium irregulare*

Growth retardation assays with the oomycete root rot pathogen *P. irregulare* were performed as described by Sohrabi et al. (2015). The purified TPS20c diterpene hydrocarbon and alcohol products were applied in triplicate experiments at concentrations of 1 μM, 10 μM, and 100 μM and the growth performance was assessed 2 days post inoculation.

### Bioinformatics and Statistical Analysis

Nucleotide and amino acid sequence alignments were performed with CLC sequence viewer 7.0 (CLC Bio) using default settings. The plastid transit peptide sequence was predicted with TargetP^3^ and ChloroP^4^. The *TPS* gene coding region sequences were acquired from TAIR 10.0^5^. Statistical differences of *Pythium* growth retardation assays were determined in R (version 3.2.1) with One-way ANOVA and *post hoc* Tukey-Kramer HSD comparisons with α ≤ 0.05.

## Results

### Enzymatic Activity of TPS20 from the Cvi Ecotype

In an effort to further characterize predominantly root-expressed *Arabidopsis* TPSs, we investigated the following *TPS* genes of the subfamily-a, which are known to be transcribed in roots based on published results and publicly available gene expression profiles (Tholl and Lee, 2011), TAIR 10.0^6^: *TPS6* (At1g70080), *TPS9* (At4g20230), *TPS19* (At3g14540), *TPS20* (At5g48110), *TPS22* (At1g33750), *TPS25* (At3g29410), *TPS26* (At1g66020), *TPS29* (At1g31950), and *TPS30* (At3g32030). With the exception of *TPS6*, we were able to clone full length cDNAs of all of these *TPS* genes from roots of the Col ecotype in the pET28a vector for functional expression in *E. coli*. While sesquiTPS and/or diTPS activities were detected for Col TPS9, TPS22, TPS25, TPS26, and TPS30 (see below), no activity was found for the full length or truncated recombinant proteins of TPS19, TPS20, and TPS29 in coexpression with (*E,E*)-FPP synthase, GGPP synthase, or *ent*-CPP synthase. We explored the possibility whether the genes encoding these proteins could be functionally active in another *Arabidopsis* ecotype. We chose the ecotype Cvi, since this ecotype has been shown to differ distinctively in its glucosinolate and volatile terpene profiles in leaves and flowers, respectively, from those of the Col ecotype (Kliebenstein et al., 2001; Tholl et al., 2005). We found that *TPS20* was also expressed in roots of the ecotype Cvi, whereas transcripts of *TPS19* and *TPS29* were not detected in the root of this ecotype (Supplementary Figure S1). We also did not observe expression of *TPS9, TPS22, TPS25*, and *TPS30* in Cvi roots.

**FIGURE 1:**
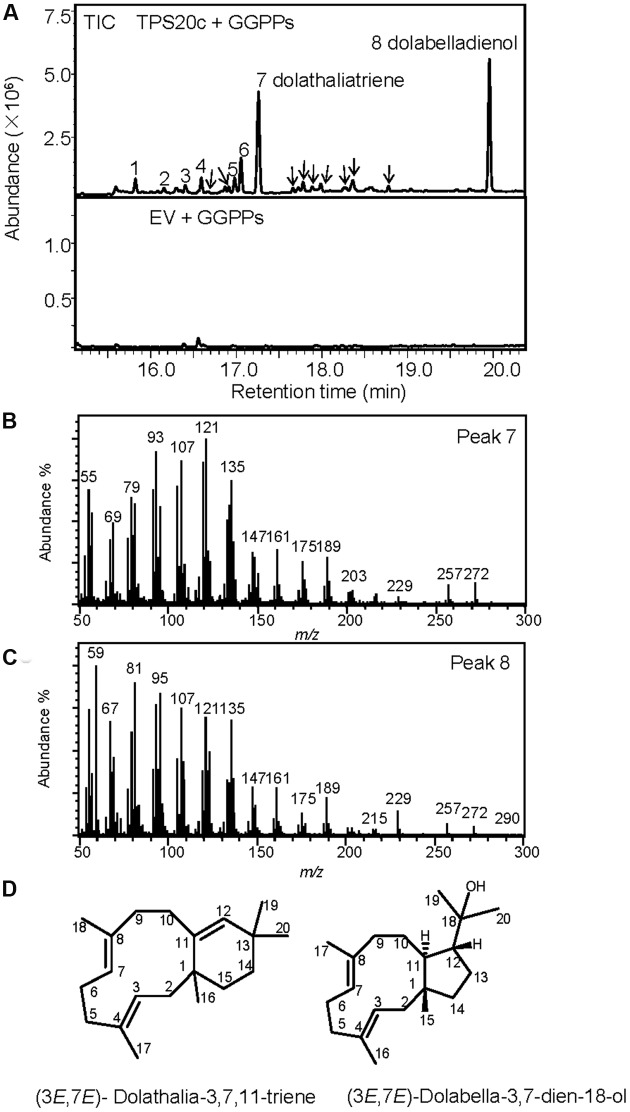
**TPS20c from Cvi functions as a dolabellane type diterpene synthase. (A)** GC-MS chromatogram of a culture extract from *Escherichia coli* coexpressing TPS20c and GGPP synthase. Ev+GGPPs, extract of *E. coli* culture expressing GGPP synthase only. Numbers indicate TPS20c products that were found *in vitro* and *in planta*. Arrows indicate other minor TPS20c diterpene products **(B,C)** Mass spectra for product 7 (dolathaliatriene) and product 8 (dolabelladienol). Mass spectra of the other numbered products are listed in Supplementary Materials (Supplementary Figure S4). **(D)** Chemical structures for dolathaliatriene and dolabelladienol.

**FIGURE 2:**
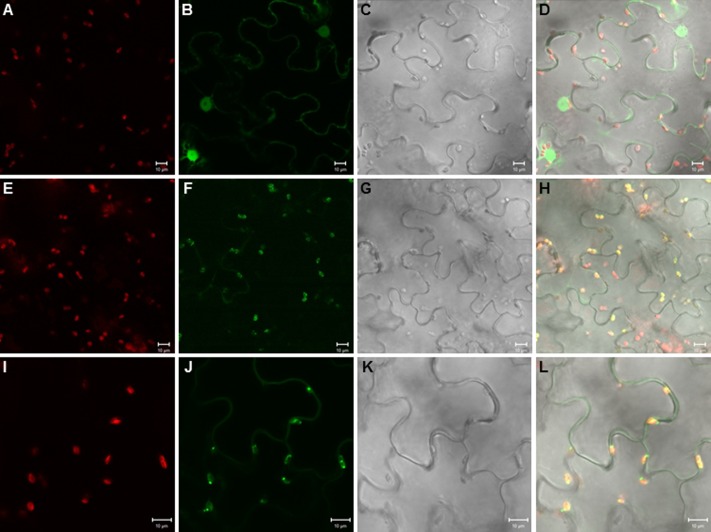
**Plastidial localization of the TPS20c protein.** Images of tobacco epidermal cells transiently expressing eGFP **(A–D)**, eGFP fused to the TPS20c full length protein **(E–H)**, and eGFP fused to the TPS20c N-terminal transit peptide **(I–L)** under the control of the CaMV 35S promoter. Images **(A,E,I)**, chlorophyll autofluorescence. Images **(B,F,J)**, eGFP fluorescence. Images **(C,G,K)**, light microscopic images. Images **(D,H,L)**, overlay of chlorophyll autofluorescence, eGFP and light microscopic images. Bar = 10 μm.

When we cloned the TPS20 cDNA from Cvi, we found several amino acid sequence difference in the Cvi TPS20 ORF in comparison to the Col TPS20 sequence (Supplementary Figure S2). These differences included an insertion of 17 amino acids in conjunction with several other amino acid substitutions in the Cvi TPS20 protein. A comparison of the Col TPS20 gene sequence with both the Col and Cvi TPS20 cDNAs indicated non-splicing of an intron (571G–622G) in the Cvi sequence (Supplementary Figure S3). The presence of this intron together with a single nucleotide deletion (675_676del) causes a major change in the TPS20 Cvi amino acid sequence between V190 and L224 (Supplementary Figures S2 and S3). To determine whether the TPS20 cDNA from Cvi would encode a functionally active protein in contrast to the inactive recombinant TPS20 protein from Col, we expressed the Cvi TPS20 protein, named TPS20c hereafter, without its putative plastidial transit peptide in *E. coli* and tested its enzymatic activity. GC-MS analysis of the TPS20c catalytic products showed that the TPS20c protein reacted with GGPP to produce several diterpene olefins and one diterpene alcohol as the major product (**Figures 1A–C**, Supplementary Figure S4). To determine the structures of these diterpenes, TPS20c was incorporated into a modular expression system, which allowed to produce sufficient amounts of the major products for purification and structure elucidation by NMR (Supplementary Figures S5–S11, Supplementary Tables S2 and S3). The predominant diterpene alcohol was identified as (3*E*,7*E*)-dolabella-3,7-dien-18-ol (**Figure 1D**). Interestingly, we found a dolabellane-related C6-C11 bicyclic scaffold for the predominant TPS20 olefin product, which, to the best of our knowledge, has not been described previously. We, therefore, named this compound, (3*E*,7*E*)-dolathalia-3,7,11-triene (**Figure 1D**). TPS20c also reacted with (*E,E*)-FPP to produce α-humulene but did not show activity in co-expression with *ent*-CPP synthase (Supplementary Figure S12). To determine the subcellular localization of the TPS20c protein, transient expression of eGFP fused N-terminally to the TPS20c full length protein or its predicted N-terminal transit peptide was performed in *N. benthamiana*. These experiments demonstrated a localization of TPS20c in plastids (**Figure 2**). Kinetic analysis of the TPS20c enzyme with GGPP as its predominant prenyl diphosphate substrate in plastids demonstrated that the catalytic activity and substrate affinity were in the range of those of class-I diTPSs characterized from *Arabidopsis* and other plant species (**Table 1**) (Hill et al., 1996; Williams et al., 2000; Vaughan et al., 2013).

**Table 1:** Steady-state kinetic constants for TPS20c.

Substrate	*K*_M_ (μM)	*V*_max_ (pkat mg^-1^)	*K*_cat_ (s^-1^)	*K*_cat_/*K*_M_ (s^-1^ μM^-1^)
GGPP	6.29 ± 0.24	12.76 ± 0.25	0.83 × 10^-3^ ±0.01 × 10^-3^	0.13 ± 0.01

### TPS20c Diterpenes in Tissues of the Cvi Ecotype

**FIGURE 3:**
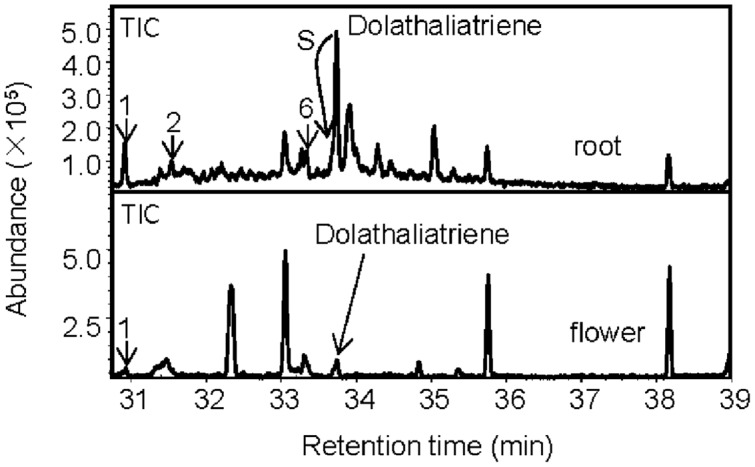
**Volatile headspace SPME-GC-MS analysis of diterpenes in *Arabidopsis* Cvi roots and flowers.** Diterpenes are marked with a number and/or arrows. The indicated compounds are identical to the enzymatic products of TPS20c (**Figure 1**). S indicates that dolathaliatriene eluted at the shoulder of another compound.

Next, we examined whether roots of the Cvi ecotype produce any of the semi-volatile TPS20c diterpene products by analyzing the headspace of root tissue by GC-MS. Several TPS20c diterpene products were detected at low levels including dolathaliatriene (**Figure 3**). Two of these compounds were also found in emissions from Cvi flowers (**Figure 3**) and trace emissions were detected in leaves.

**FIGURE 4:**
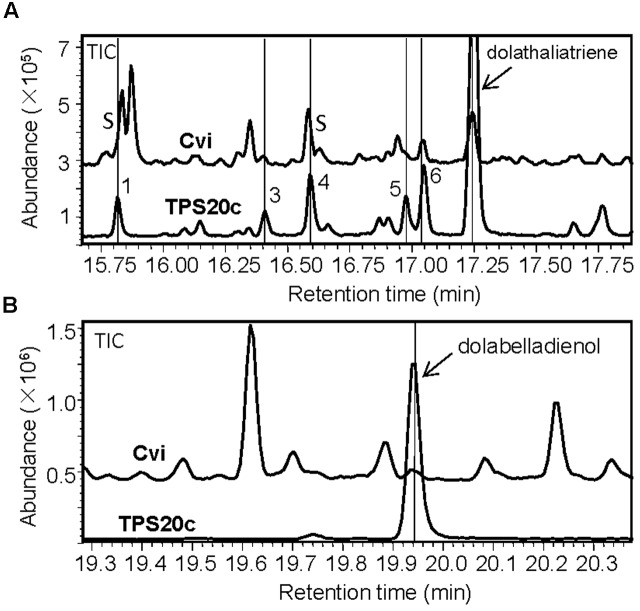
**GC-MS comparison of diterpenes produced by TPS20c and Cvi plants. (A)** Hexane extracts of a whole Cvi plant (flowers, leaves, roots) and TPS20c enzymatic products. TPS20c diterpene products that were detected in the plant extract are marked with lines and numbers. S indicates that diterpene compounds eluted at the shoulder of another compound. **(B)** Ethyl acetate extracts of a Cvi plant and TPS20c enzymatic products. The dolabelladienol peak is marked with a line and arrow.

Comparative GC-MS analysis of hexane extracts of whole flowering Cvi plants and the diterpene olefins produced by TPS20c *in vitro* showed that several of the TPS20c products were identical to the plant produced diterpene compounds based on their retention times and mass spectra (**Figure 4A**). Further analysis of ethyl acetate extracts of Cvi plants revealed the presence of dolabelladienol as another enzymatic product of TPS20c (**Figure 4B**). Hence, TPS20c was demonstrated to be involved in the formation of the detected diterpenes in the Cvi ecotype. The detection of the TPS20 products throughout the Cvi plant is consistent with the expression of *TPS20c* in flowers, leaves, and roots of this ecotype (Supplementary Figure S1B). Quantitative analysis of dolathaliatriene in hexane extracts of Cvi whole flowering plants indicated a concentration of approximately 150 ng/g FW based on calibration with the pure compound.

**FIGURE 5:**
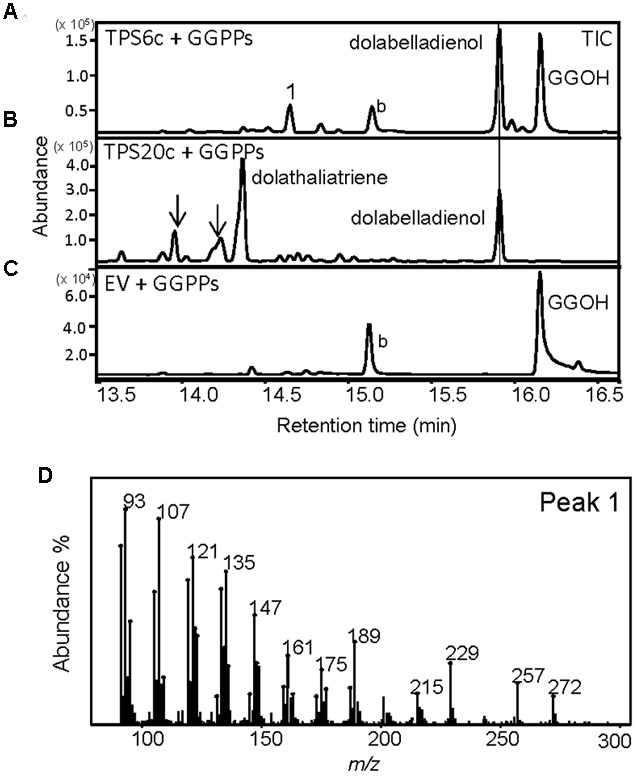
**TPS6c reacts with GGPP to produce dolabelladienol and an unknown diterpene.** GC-MS chromatograms of culture extracts from *E. coli* coexpressing truncated TPS6c **(A)** or TPS20c **(B)** with GGPP synthase. Dolabelladienol is indicated with a line. Arrows indicate other TPS20c diterpene products. **(C)** Extract of *E. coli* culture expressing GGPP synthase only. EV, empty vector; GGOH, geranylgeraniol; b, background. **(D)** The mass spectrum of the unknown diterpene product is depicted [peak 1 in **(A)**].

We did not find any α-humulene, the *in vitro* sesquiterpene product of TPS20c, *in planta* indicating that TPS20c functions as a diTPS *in vivo*. The TPS20c diterpene compounds have not been detected in Col-0, which is in agreement with the presence of a non-functional TPS20 allele in this ecotype. Besides TPS20c, we found that the N-terminally truncated recombinant protein encoded by the gene *TPS6* (TPS6c), which is expressed at low levels in Cvi roots (Supplementary Figure S1), also produces dolabelladienol besides another unidentified diterpene olefin (**Figure 5**).

### Effects of Dolabellane Type Diterpenes on the Growth of *Pythium irregulare*

**FIGURE 6:**
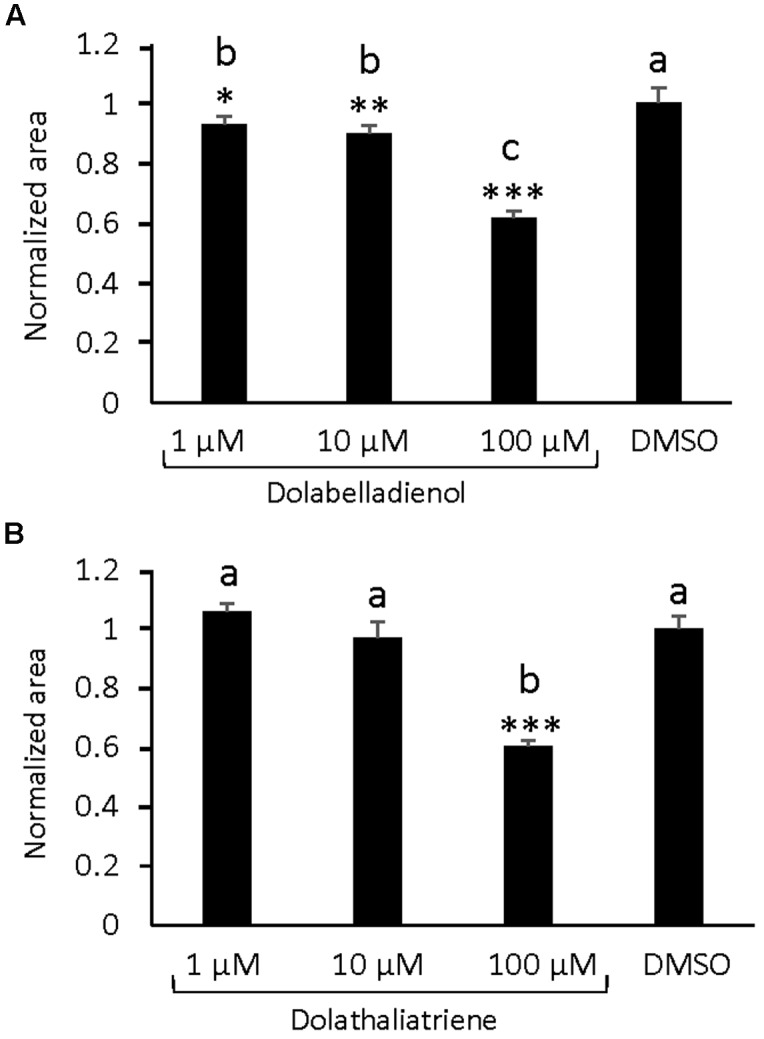
***In vitro* effects of TPS20c diterpene products on the growth of *Pythium irregulare*. (A)** Growth inhibition assays with different concentration of dolabelladienol. **(B)** Growth inhibition assays with different concentration of dolathaliatriene. Values represent the mean ± standard error mean (SEM) of 3 biological replicates. Normalized area was calculated by dividing two averaged radial measurements (area = πr_avg_^2^) of the growth area by the average area of the mock (DMSO). Statistical differences were determined in R (version 3.2.1) by One-way ANOVA and *post hoc* Tukey-Kramer HSD comparisons against the mock where α ≤ 0.05. ^∗^ = *p* ≤ 0.05, ^∗∗^ = 0.05 ≥*p* ≥ 0.001, ^∗∗∗^ = *p* ≤ 0.0001.

Dolabellane type diterpenes have been reported to have antimicrobial activity (Ioannou et al., 2011). Since dolathaliatriene as the major TPS20 diterpene product was detected at concentrations lower than those found for rhizathalenes (Vaughan et al., 2013), we considered it to be difficult to conduct statistically robust bioassays *in vivo*. Moreover, we could not find any induction of *TPS20c* gene expression by treatment with the defense hormone jasmonic acid (Supplementary Figure S1C). Nevertheless, we tested whether the TPS20c diterpene products could affect the growth of root microbial pathogens by performing *in vitro* growth retardation assays with the root rot pathogen *P. irregulare*, which causes mild disease symptoms in roots of wild type *Arabidopsis* plants (Sohrabi et al., 2015). Previous studies showed that the volatile homoterpene (*E*)-4,8-dimethyl-1,3,7-nonatriene (DMNT) reduced *Pythium* mycelium growth rate by 30% at concentrations as low as 10 nM (Sohrabi et al., 2015). *In vitro* assays with different concentrations of the terpene compounds demonstrated significant effects at concentrations of 1 μM (∼290 ng/mL), 10 μM (2.9 μg/mL), and 100 μM (29 μg/mL) for dolabelladienol with a growth reduction of 8, 10, and 40%, respectively. For dolathaliatriene, a significant reduction of *Pythium* growth was only observed at a concentration of 100 μM (27 μg/mL) (**Figures 6A,B**).

### Enzymatic Activities of Other Root-expressed TPSs of the Col Ecotype

Besides TPS20c and TPS6c, we were able to find enzymatic activity for proteins encoded by five so far uncharacterized *TPS* genes with expression in roots of the Col ecotype. Unfortunately, because of the lower activity of the expressed enzymes, it was not possible to obtain sufficient amounts of purified compounds for NMR analysis and structural elucidation of the diterpene products of these enzymes.

**FIGURE 7:**
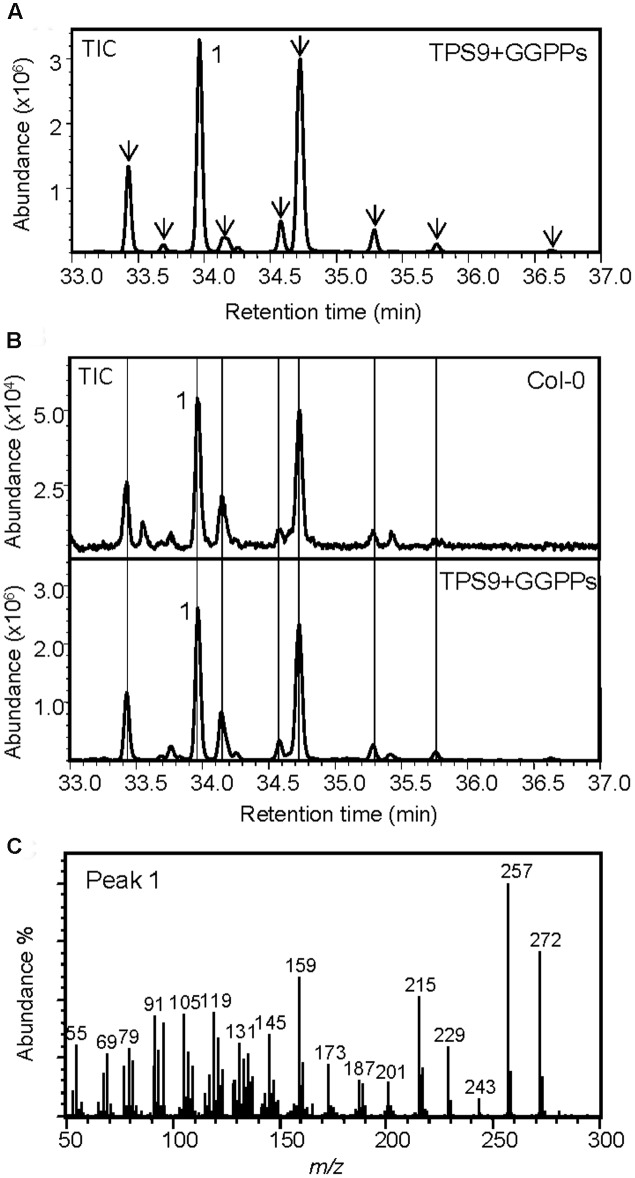
**TPS9 from the Col ecotype functions as a diterpene synthase. (A)** GC-MS chromatogram of a culture extract from *E. coli* coexpressing TPS9 and GGPP synthase. **(B)** GC-MS detection of diterpenes produced by TPS9 in the Col root. Diterpenes extracted from root tissue were compared with TPS9 diterpene products made from recombinant expression as labeled with lines. **(C)** Mass spectrum of the major product 1. Mass spectra of minor TPS9 diterpene products (marked with arrows in **A**) are listed in Supplementary Materials (Supplementary Figure S13).

The recombinant protein of TPS9 exhibited diTPS activity in co-expression with GGPP synthase but not *ent*-CPP synthase in *E. coli*. TPS9 converted GGPP to one predominant diterpene olefin and eight minor diterpene products, none of which could be identified by mass spectral comparisons (**Figures 7A,C**; Supplementary Figure S13). By applying a more efficient protocol of organic solvent extraction and partial compound purification (see Materials and Methods) in comparison to the previously used hexane-based protocol (Vaughan et al., 2013), we could detect most of the diterpene products of TPS9 in Col roots at very low levels (**Figure 7B**). When co-expressed with (*E,E*)-FPP synthase, TPS9 produced trace amounts of nerolidol and (*E*)-β-farnesene; these products were not detected in extracts of Col roots.

**FIGURE 8:**
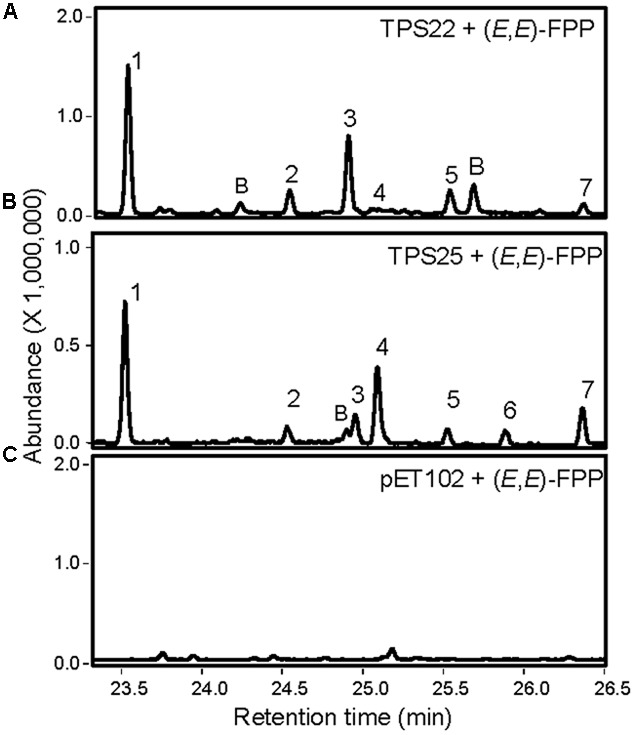
**Analysis of products formed by recombinant TPS22 and TPS25 enzymes from (*E,E*)-FPP using SPME-GC-MS.** TPS22 and TPS25 proteins without the N-terminal transit peptide were used for enzyme activity assays. **(A,B)** GC-MS chromatograms of enzymatic products formed by recombinant TPS22 and TPS25. Sesquiterpene products were identified by comparison with authentic standards (1 and 7) or by library suggestion (for 2, 3, 4, 5, and 6). 1, (*E*)-β-farnesene; 2 and 3, α-farnesene isomers; 4, β-bisabolene; 5 and 6, bisabolene isomers; 7, (*E*)-nerolidol; B: background. **(C)** GC-MS analysis of products formed by extracts of *E. coli* expressing the pET102 vector without an insert.

The remaining TPS proteins showed TPS activities; however, we were unable to detect their enzymatic products in Col roots. Recombinant truncated TPS26 protein produced 9 unknown diterpene olefins in co-expression with GGPP synthase (Supplementary Figure S14). The truncated recombinant protein encoded by gene *TPS30* reacted with GGPP to form two unknown diterpenes (Supplementary Figures S15A,B). Further analysis revealed that TPS30 converted *ent*-CPP to *ent*-pimara-8(14),15-diene and *ent*-manool (Supplementary Figure S15C). TPS26 and TPS30 also exhibited sesquiTPS activities with trace amounts of α-humulene and (*E*)-β-farnesene being produced by TPS26 and traces of nerolidol and (*E*)-β-farnesene being produced by TPS30. Finally, the recombinant proteins of genes *TPS22* and *TPS25* did not show any diTPS activities. Instead, both proteins converted (*E,E*)-FPP into sesquiterpenes *in vitro* with (*E*)-β-farnesene as the main compound (**Figure 8**; Huh, 2011).

## Discussion

### *Arabidopsis* TPS20 and Other Root-Expressed TPSs of the Subfamily-a Have Class-I Diterpene Synthase Activities

To date, only a single root-specific diTPS named rhizathalene synthase (TPS8) in the TPS-a type clade of the *Arabidopsis* TPS family has been characterized (Vaughan et al., 2013). Our results show that TPS20 and other enzymes of this clade, whose genes are partly or exclusively expressed in roots, also exhibit diTPS activity. DiTPS in the TPS-a subfamily belong to the βα-domain class I enzymes that catalyze the formation of their products by dephosphorylation of the C20-substrates GGPP or CPP and subsequent rearrangements of the resulting intermediate carbocations. In contrast, class II enzymes with a γβα-domain architecture facilitate a protonation-dependent cyclization of GGPP to CPP-type bicyclic diphosphate products (Zi et al., 2014). Only a single TPS (TPS30) of the class I enzymes we tested could convert both GGPP and *ent*-CPP (the product of the *ent*-CPP synthase TPS31, Sun and Kamiya, 1997) into diterpene products, suggesting a limited C20-substrate promiscuity among the analyzed diTPSs.

We were able to detect the dolabellane type diterpenes produced by TPS20 and in part by TPS6 as well as the enzymatic products of TPS9 *in planta*. The detection of the TPS9 diterpenes in root tissue of the Col ecotype required a more efficient extraction protocol (see Materials and Methods) since these compounds had not been observed with previous extraction procedures (Vaughan et al., 2013). The products of the other functionally active diTPSs (TPS26, TPS30) remained undetected. It is possible that the compounds produced by these enzymes undergo further *in vivo* conversions to non-volatile derivatives. TPS26 and the closely related TPS9 are part of a co-expression network that includes the cytochrome P450 monooxygenase CYP78A8 (ATTED-II, CYPedia). Therefore, it is possible that the diterpene products of TPS26 and maybe of TPS9 are further converted by hydroxylation and other subsequent modifications.

Although TPS20c exhibits sesquiTPS activity *in vitro*, we could not find its sesquiterpene product α-humulene *in vivo*. The finding suggests that the sequiTPS activity is negligible *in vivo* because of the limited concentration of (*E,E*)-FPP in plastids. A similar scenario can be assumed for TPS22 and TPS25, which are presumably located in plastids but produce only sesquiterpenes with β-farnesene as the main compound *in vitro*. Accordingly, we have detected only trace amounts of β-farnesene (by SPME) in root tissue. However, it cannot be excluded that these enzymes as well as TPS19 and TPS29, which are positioned in the same branch of the type-a clade as TPS22 and TPS25 and are inactive *in vitro*, have other functions by possibly accepting longer prenyl diphosphates as substrates.

.

**FIGURE 9:**
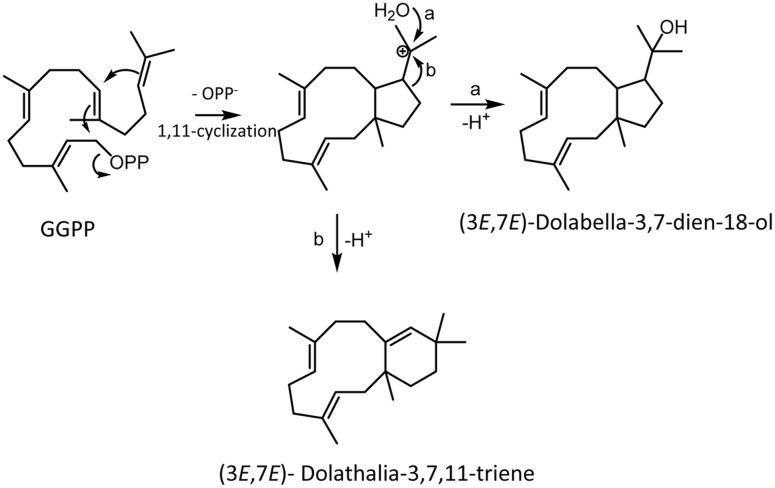
**Proposed reaction mechanism of TPS20c**.

TPS-a type clades with expanded clusters of diTPSs have been found in several other angiosperms. For example, a divergence of diTPS sequences has been described for cembrene synthase like enzymes in the TPS families of Euphorbiaceae (*Euphorbia peplus, Jatropha gossypiifolia*) and Celastraceae (*Tripterygium wilfordii*) (Zerbe et al., 2013). Interestingly, phylogenetic comparisons show that the *Arabidopsis* TPS-a type diTPSs are positioned together with these diterpene macrocyclases in a clade more closely related to TPS-a type class I gymnosperm diTPSs and sesquiTPSs (Zerbe and Bohlmann, 2015). The ability of TPS20 and the closely related TPS6 from Cvi, to form macrocyclic diterpenes (**Figure 9**) supports the functional relatedness to the diTPSs in this group. The reaction facilitated by TPS20c presumably proceeds via dephosphorylation of GGPP and the formation of a dolabelanyl carbocation, which is then converted to dolathaliatriene by a Wagner-Meerwein rearrangement and deprotonation or directly to dolabelladienol by quenching with water (**Figure 9**). Enzymes that enable oxygenation by water quenching have been reported from a variety of other class I and II diTPSs (Zerbe and Bohlmann, 2015). It remains to be determined whether the products of the other characterized diTPSs are also of macrocyclic nature.

### Diterpene Biosynthesis in *Arabidopsis* Occurs in an Ecotype Specific Manner

In the Col ecotype more than half of the genes of the TPS family including 12 genes of the TPS-a type clade are partly or exclusively expressed in roots (Tholl and Lee, 2011). Several of the terpene products that have been associated with these genes are almost exclusively found in the Col root tissue such as the TPS23/27 monoterpene product 1,8-cineole (Chen et al., 2004), the rhizathalene diterpenes made by TPS8 (Vaughan et al., 2013), *cis*-γ-bisabolene produced by TPS12/13 (Ro et al., 2006), or the diterpene products of TPS9 detected in this work. By contrast, monoterpene or sesquiterpene products of the TPSs that are expressed constitutively or in response to biotic stress in leaves and flowers (Tholl and Lee, 2011) are largely absent in root tissue, which suggests functional adaptations in terpene production to above and belowground environments. However, this tissue-specific distinction is ecotype dependent since the dolabellane type diterpenes made by TPS20 in Cvi are produced throughout the plant. The TPS20c diterpenes appear to have been selectively favored by the Cvi ecotype, a notion that is also supported by the observation that several of the examined type-a TPSs are not expressed in Cvi roots (Supplementary Figure S1).

TPS20c diterpenes are absent in the Col ecotype because of a non-functional TPS20 allele in this ecotype. This finding supports previous studies in *Arabidopsis*, which demonstrated that *TPS* allelic differences contribute to the ecotype-specific natural variation of constitutive and herbivore-induced volatile terpene biosynthesis in flowers and leaves (Tholl et al., 2005; Huang et al., 2010). Allelic variation and pseudogenization are common among defense metabolic pathways, especially in gene pools of the large *TPS* gene families, which are subject to increased diversification and turnover under varying selection pressures (Kliebenstein et al., 2001; Thibaud-Nissen et al., 2009; Zou et al., 2009; Zhuang et al., 2012; Warren et al., 2015). Together, our results exemplify that sequence divergence in the TPS-a subfamily paired with functional differentiation leads to plasticity in the “terpene landscape” of *Arabidopsis* ecotypes above and belowground.

### Dolabellane Type Diterpenes Occur in Different Organisms and Exhibit Defensive Activities

Dolabellane type diterpenes have been detected primarily in marine animals and algae. For example, representatives of these macrocyclic diterpenes were isolated from the sea hare (mollusk) *Dolabella californica* (Ireland et al., 1976), Gorgonian octocorals of the genus *Eunicea* (Wei et al., 2010) and brown algae (Viano et al., 2009; Ioannou et al., 2011). Among land plants, dolabellanes have been reported from liverworts and only a single study detected dolabellane compounds in higher land plants in the “Chinese perfume plant” *Aglaia odorata* (Cai et al., 2005; Cai et al., 2010). The occurrence of this class of diterpenes in animals and plants raises the question of conserved biological functions of dolabellane diterpenes. Bioactivity assays have shown that dolabellanes exhibit antimicrobial and antiprotozoan activities (Viano et al., 2009; Wei et al., 2010; Ioannou et al., 2011). We tested equivalent defensive activities against the root microbial pathogen *P. irregulare*. Although we observed a dose-dependent reduction of mycelium growth in the presence of the TPS20c products, the effective *in vitro* concentrations were higher than the level of the major TPS20 diterpene compound dolathaliatriene *in planta* (∼150 ng/g FW). It can be assumed that the direct products of TPS20 have no major effect on *Pythium* growth *in vivo* under physiological conditions.

Plant derived diterpenes have been implicated with different biotic and abiotic activities as allelopathic compounds, phytoalexins, herbivore deterrents, or in the context of drought tolerance (Jassbi et al., 2010; Schmelz et al., 2011; Xu et al., 2012; Vaughan et al., 2013, 2015). In *Arabidopsis*, the observation of low or trace levels of diverse diterpene products, especially in roots, paired with the finding of an expanded TPS-a clade of diTPSs raises questions about the significance of terpenoid biochemical promiscuity and plasticity and its possible response to selective pressures. The detected semi-volatile compounds or their non-volatile downstream derivatives may, even at low levels, function synergistically or have additive effects in interactions with microbes or other target organisms in the rhizosphere or endosphere. Moreover, the compounds may exhibit signaling functions at picomolar concentrations as was demonstrated for the diterpenoid dehydroabietinal as a vascular signal in *Arabidopsis* systemic acquired resistance (Chaturvedi et al., 2012).

## Author Contributions

QW, MJ, J-HH, AM, and DT conceived and designed the experiments and conducted the work. QW, MJ, J-HH, AM, RP, and DT interpreted the data and QW, MJ, RP, and DT wrote the paper.

## Conflict of Interest Statement

The authors declare that the research was conducted in the absence of any commercial or financial relationships that could be construed as a potential conflict of interest.

## Footnotes

1 www.Arabidopsis.org

2 http://www.brc.riken.jp/lab/epd/catalog/cdnaclone.html

3 http://www.cbs.dtu.dk/services/TargetP/

4 http://www.cbs.dtu.dk/services/ChloroP/

5 www.Arabidopsis.org

6 www.Arabidopsis.org
