# Supplementary material for: Identification of a Dolabellane Type Diterpene Synthase and other Root-Expressed Diterpene Synthases in Arabidopsis
Source: Front Plant Sci. 2016 Nov 25;7:1761. doi: 10.3389/fpls.2016.01761 (PMC5122590; doi:10.3389/fpls.2016.01761)
Supplement: Supplementary file 1 [file Data_Sheet_1.pdf]

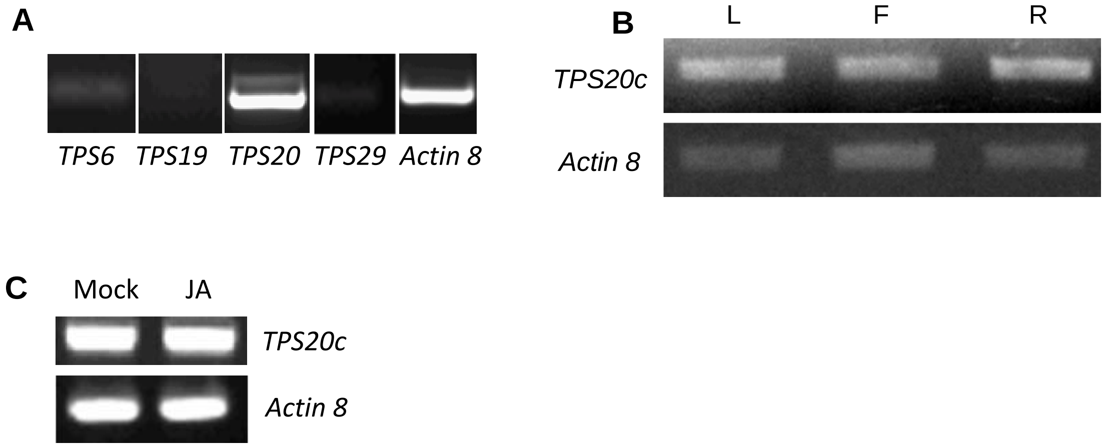

Figure S1. *TPS* gene expression in Cvi. (A) Expression of selected *TPS* genes in the Cvi root. (B) *TPS20c* gene expression in Cvi tissues. L: leaf; F: flower; R: root.
(C) *TPS20c* gene expression in axenically grown roots of Cvi upon 24 h of treatment with 100 µM jasmonic acid.

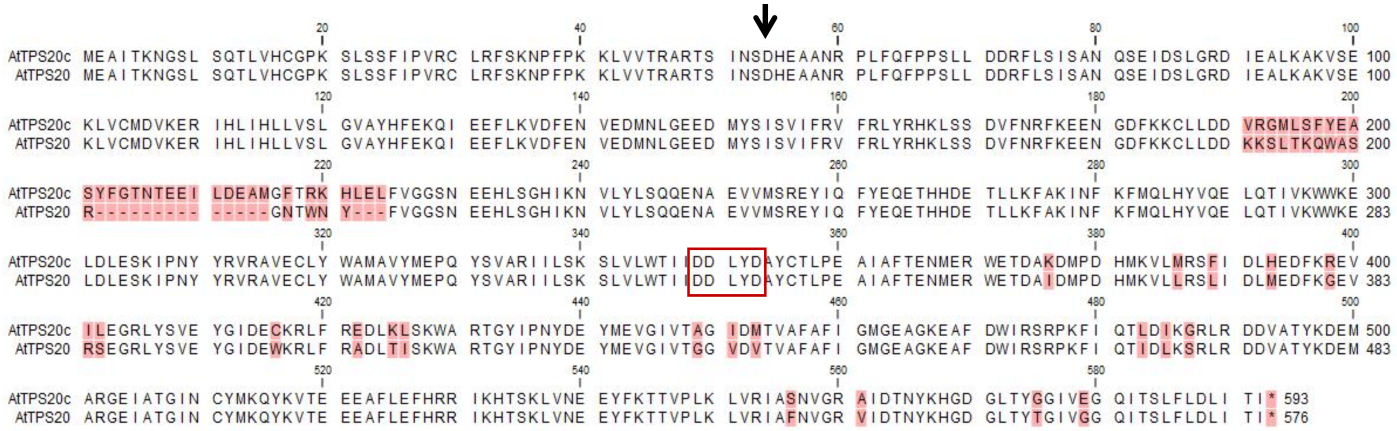

Figure S2. Amino acid sequence alignment of TPS20 (Col ecotype) and TPS20c (Cvi ecotype). Amino acid insertions and substitutions are highlighted in pink. The DDXXD motif is indicated with a red box. The arrow shows the position of the truncation of the transit peptide for protein expression in *E.coli* and transit peptide fusion to eGFP for transient expression in tobacco leaves.

```
                                    20                    40                    60                    80                   100
AtTPS20c  ATGGAAGCAA TAACTAAAAA TGGGTCGCTC TCTCAAACTC TTGTTCATTG TGGGCCAAAA AGCTTGAGCT CGTTCATCCC AGTTCGCTGT CTTAGGTTTT 100
AtTPS20   ATGGAAGCAA TAACTAAAAA TGGGTCGCTC TCTCAAACTC TTGTTCATTG TGGGCCAAAA AGCTTGAGCT CGTTCATCCC AGTTCGCTGT CTTAGGTTTT 100
                                   120                   140                   160                   180                   200
AtTPS20c  CTAAGAATCC GTTCCCCAAA AAGTTGGTCG TAACTAGAGC TAGGACGAGC ATTAACAGTG ATCATGAAGC CGCAAATCGT CCGTTATTTC AGTTTCCACC 200
AtTPS20   CTAAGAATCC GTTCCCCAAA AAGTTGGTCG TAACTAGAGC TAGGACGAGC ATTAACAGTG ATCATGAAGC CGCAAATCGT CCGTTATTTC AGTTTCCACC 200
                                   220                   240                   260                   280                   300
AtTPS20c  TTCCCTATTG GATGATCGCT TCCTTTCCAT TTCCGCCAAC CAGTCGGAAA TTGATAGCCT TGGGAGAGAT ATCGAGGCAC TTAAGGCTAA AGTGAGTGAA 300
AtTPS20   TTCCCTATTG GATGATCGCT TCCTTTCCAT TTCCGCCAAC CAGTCGGAAA TTGATAGCCT TGGGAGAGAT ATCGAGGCAC TTAAGGCTAA AGTGAGTGAA 300
                                   320                   340                   360                   380                   400
AtTPS20c  AAGCTGGTTT GTATGGACGT AAAGGAGAGG ATTCACTTGA TCCATCTGCT AGTCAGTCTT GGAGTCGCTT ACCATTTCGA GAAACAGATC GAAGAATTCC 400
AtTPS20   AAGCTGGTTT GTATGGACGT AAAGGAGAGG ATTCACTTGA TCCATCTGCT AGTCAGTCTT GGAGTCGCTT ACCATTTCGA GAAACAGATC GAAGAATTCC 400
                                   420                   440                   460                   480                   500
AtTPS20c  TGAAAGTTGA TTTCGAGAAC GTGGAGGATA TGAACCTGGG AGAAGAGGAT ATGTATAGCA TTTCTGTCAT CTTCAGGGTT TTTAGGCTGT ACCGTCACAA 500
AtTPS20   TGAAAGTTGA TTTCGAGAAC GTGGAGGATA TGAACCTGGG AGAAGAGGAT ATGTATAGCA TTTCTGTCAT CTTCAGGGTT TTTAGGCTGT ACCGTCACAA 500
                                   520                   540                   560                   580                   600
AtTPS20c  GTTATCTTCC GATGTGTTCA ATAGGTTTAA AGAAGAAAAT GGGGATTTTA AGAAATGCCT TCTCGATGAT GTAAGAGGTA TGCTAAGCTT CTACGAAGCT 600
AtTPS20   GTTATCTTCC GATGTGTTCA ATAGGTTTAA AGAAGAAAAT GGGGATTTTA AGAAATGCCT TCTCGATGAT ---------- ---------- ---------- 570
                                   620                   640                   660                   680                   700
AtTPS20c  TCATATTTTG GGACGAATAC AGAAGAAATC CTTGACGAAG CAATGGGCTT CACGCGGAAA CACTTGGAAC TATTT-GTTG GAGGCAGCAA TGAGGAACAT 699
AtTPS20   ---------- ---------- --AAGAAATC CTTGACGAAG CAATGGGCTT CACGCGGAAA CACTTGGAAC TATTTTGTTG GAGGCAGCAA TGAGGAACAT 648
                                   720                   740                   760                   780                   800
AtTPS20c  TTATCTGGGC ACATAAAAAA CGTGTTGTAT CTTTCTCAGC AAGAAAATGC AGAGGTGGTG ATGTCAAGAG AATATATACA GTTCTATGAA CAAGAGACAC 799
AtTPS20   TTATCTGGGC ACATAAAAAA CGTGTTGTAT CTTTCTCAGC AAGAAAACGC AGAGGTGGTG ATGTCAAGAG AATATATACA GTTCTATGAA CAAGAGACAC 748
                                   820                   840                   860                   880                   900
AtTPS20c  ATCACGACGA GACCTTGCTA AAATTTGCCA AGATCAATTT CAAGTTCATG CAGTTGCATT ATGTTCAAGA GCTTCAAACT ATCGTGAAAT GGTGGAAAGA 899
AtTPS20   ATCACGACGA GACCTTGCTA AAATTTGCCA AGATCAATTT CAAGTTCATG CAGTTGCATT ATGTTCAAGA GCTTCAAACT ATCGTGAAAT GGTGGAAAGA 848
                                   920                   940                   960                   980                 1,000
AtTPS20c  GCTTGACTTG GAATCAAAGA TCCCTAATTA CTACAGAGTT AGAGCCGTGG AGTGCCTCTA CTGGGCAATG GCAGTGTATA TGGAGCCACA ATATTCAGTT 999
AtTPS20   GCTTGACTTG GAATCAAAGA TCCCTAATTA CTACAGAGTT AGAGCCGTGG AGTGCCTCTA CTGGGCAATG GCAGTGTATA TGGAGCCACA ATATTCAGTT 948
                                 1,020                 1,040                 1,060                 1,080                 1,100
AtTPS20c  GCTAGAATAA TACTGAGCAA GTCCTTGGTG TTGTGGACCA TTATAGATGA CCTATATGAT GCCTATTGTA CTCTTCCAGA AGCTATTGCT TTCACTGAAA 1099
AtTPS20   GCTAGAATAA TACTGAGCAA GTCCTTGGTG TTGTGGACCA TTATAGATGA CCTATATGAT GCCTATTGTA CTCTTCCAGA AGCTATTGCT TTCACTGAAA 1048
                                 1,120                 1,140                 1,160                 1,180                 1,200
AtTPS20c  ATATGGAAAG GTGGGAAACT GATGCCAAAG ACATGCCAGA TCATATGAAG GTCCTCATGA GGTCATTCAT AGACCTTCAC GAAGATTTCA AACGAGAAGT 1199
AtTPS20   ATATGGAAAG GTGGGAAACT GATGCCATAG ACATGCCAGA TCATATGAAG GTCCTCTTGA GGTCATTGAT AGACCTTATG GAAGATTTCA AAGGAGAAGT 1148
                                 1,220                 1,240                 1,260                 1,280                 1,300
AtTPS20c  GATTTTAGAA GGGAGATTGT ACAGTGTGGA ATATGGAATA GACGAGTGTA AAAGATTATT CAGAGAGGAC CTTAAACTAT CAAAGTGGGC ACGCACGGGA 1299
AtTPS20   GAGATCAGAA GGAAGATTGT ACAGTGTGGA ATATGGAATA GACGAGTGGA AAAGACTATT CAGAGCGGAC CTTACAATAT CAAAGTGGGC ACGCACGGGA 1248
                                 1,320                 1,340                 1,360                 1,380                 1,400
AtTPS20c  TACATACCAA ACTATGACGA ATACATGGAG GTTGGAATAG TGACTGCGGG AATAGATATG ACGGTTGCGT TCGCCTTCAT TGGTATGGGG GAGGCAGGAA 1399
AtTPS20   TACATACCAA ACTATGACGA ATACATGGAG GTTGGAATAG TGACTGGGGG AGTAGATGTC ACGGTTGCGT TCGCCTTCAT TGGTATGGGG GAAGCAGGAA 1348
                                 1,420                 1,440                 1,460                 1,480                 1,500
AtTPS20c  AAGAAGCTTT TGACTGGATA AGATCAAGAC CGAAATTCAT TCAAACTCTA GATATAAAAG GACGTCTCAG AGATGATGTT GCCACCTACA AGGATGAAAT 1499
AtTPS20   AAGAAGCTTT TGACTGGATA AGATCAAGAC CGAAATTCAT TCAAACTATA GATCTAAAAT CACGTCTCAG AGATGATGTT GCCACCTACA AGGATGAAAT 1448
                                 1,520                 1,540                 1,560                 1,580                 1,600
AtTPS20c  GGCTAGAGGA GAGATTGCGA CAGGAATCAA CTGCTATATG AAACAATATA AAGTTACCGA AGAAGAAGCG TTTCTAGAGT TTCATAGAAG GATTAAACAT 1599
AtTPS20   GGCTAGAGGA GAGATTGCGA CAGGAATCAA CTGCTATATG AAACAATATA AAGTTACCGA AGAAGAAGCG TTTCTAGAGT TTCATAGAAG GATTAAACAT 1548
                                 1,620                 1,640                 1,660                 1,680                 1,700
AtTPS20c  ACTTCTAAGC TAGTGAATGA AGAGTATTTT AAGACGACAG TACCTTTGAA ACTTGTGCGT ATAGCTTCTA ACGTTGGACG GGCTATCGAT ACCAACTACA 1699
AtTPS20   ACTTCCAAGC TAGTGAATGA AGAGTATTTT AAGACGACAG TACCTTTGAA ACTTGTGCGT ATAGCTTTTA ACGTTGGACG GGTTATCGAT ACCAACTACA 1648
                                 1,720                 1,740                 1,760                 1,780
AtTPS20c  AGCATGGCGA TGGATTAACC TATGGAGGGA TAGTAGAAGG CCAGATCACC TCTTTATTCC TTGATCTAAT CACCATTTAG 1779
AtTPS20   AGCATGGCGA TGGATTAACC TATACAGGGA TAGTAGGAGG CCAAATCACC TCTTTGTTCC TTGATCTAAT CACCATTTAG 1728
```

Figure S3. Nucleotide sequence alignment of *TPS20* (ecotype Col) and *TPS20c* (ecotype Cvi). The *TPS20c* sequence has a single nucleotide deletion (675_676del) and a 52 bp insertion (571G to 622G) in comparison to the *TPS20* sequence. Nucleotide insertions, deletions, and substitutions are highlighted in pink.

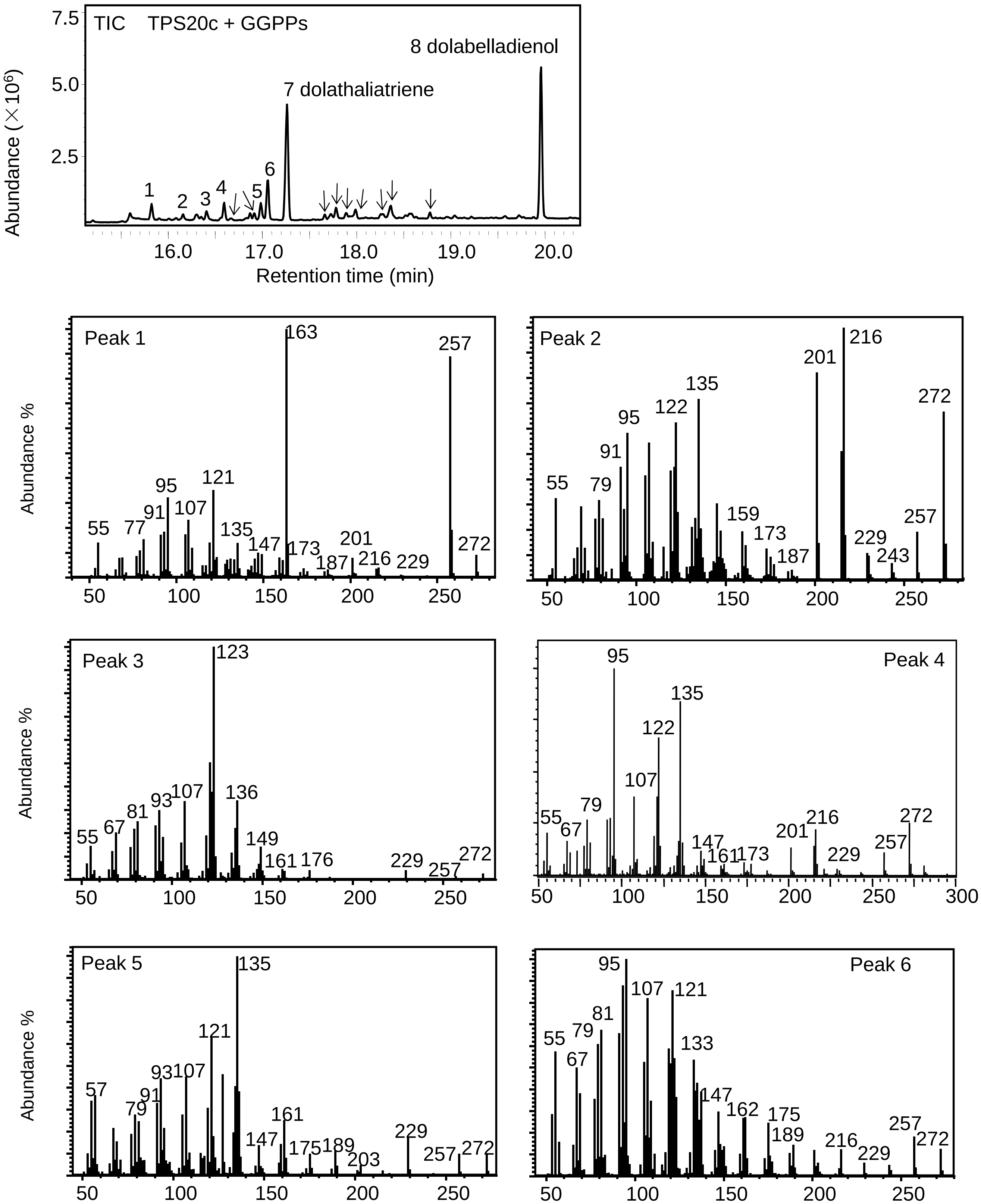

Figure S4. Mass spectra of minor diterpene products 1-6 of TPS20c.

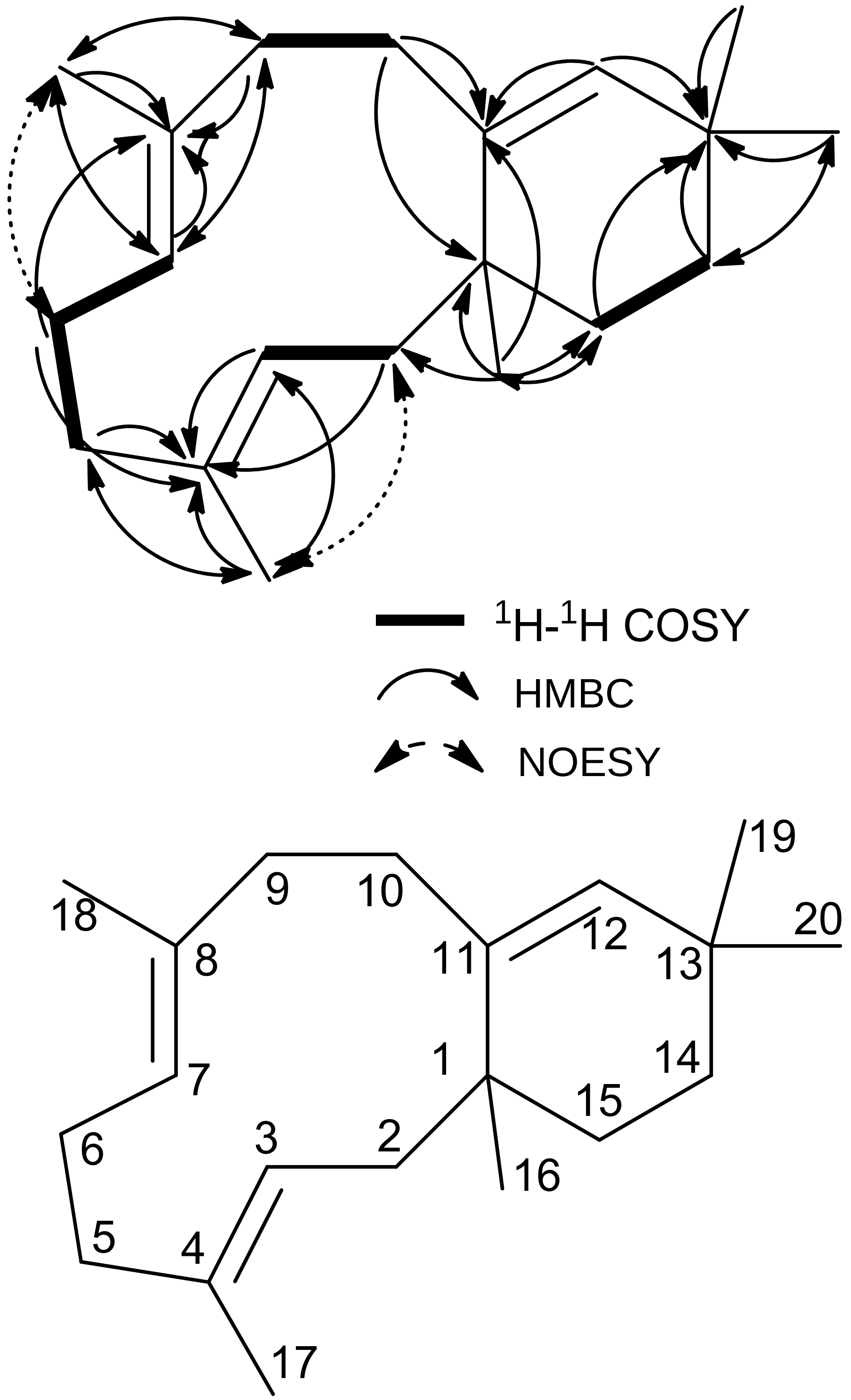

Figure S5. Important correlations of COSY, HMBC and NOESY observed for the major diterpene product of TPS20c.

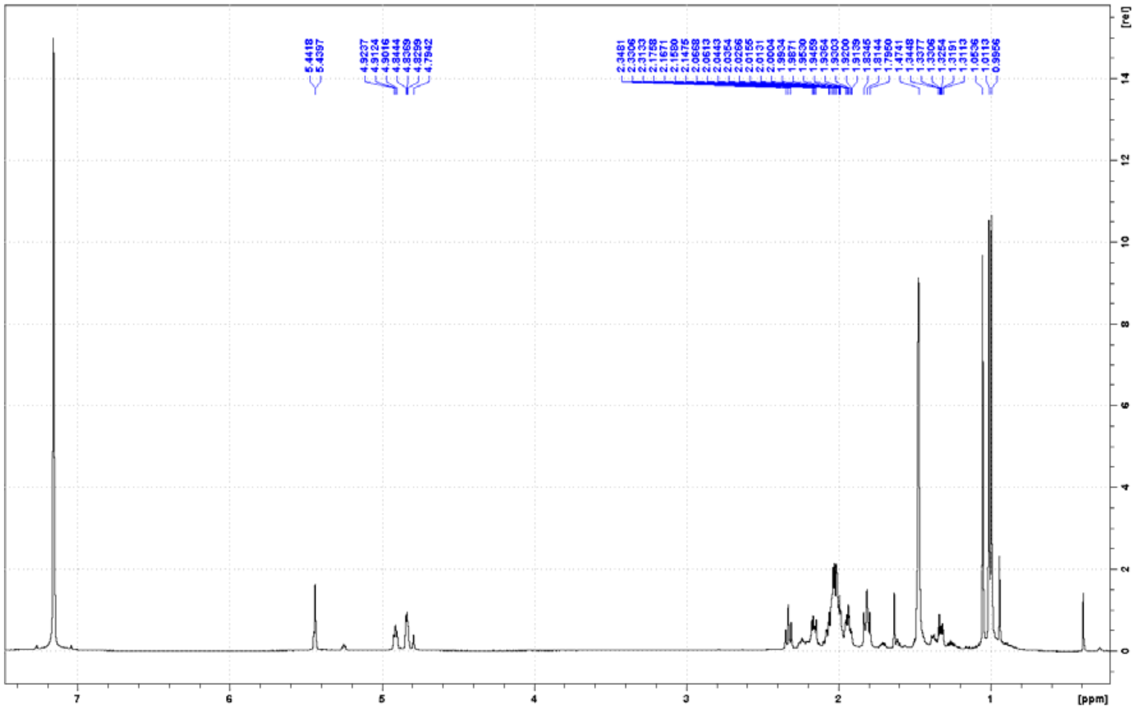

Figure S6. ^{1}H spectrum of the TPS20c major diterpene compound (solvent C_6D_6).

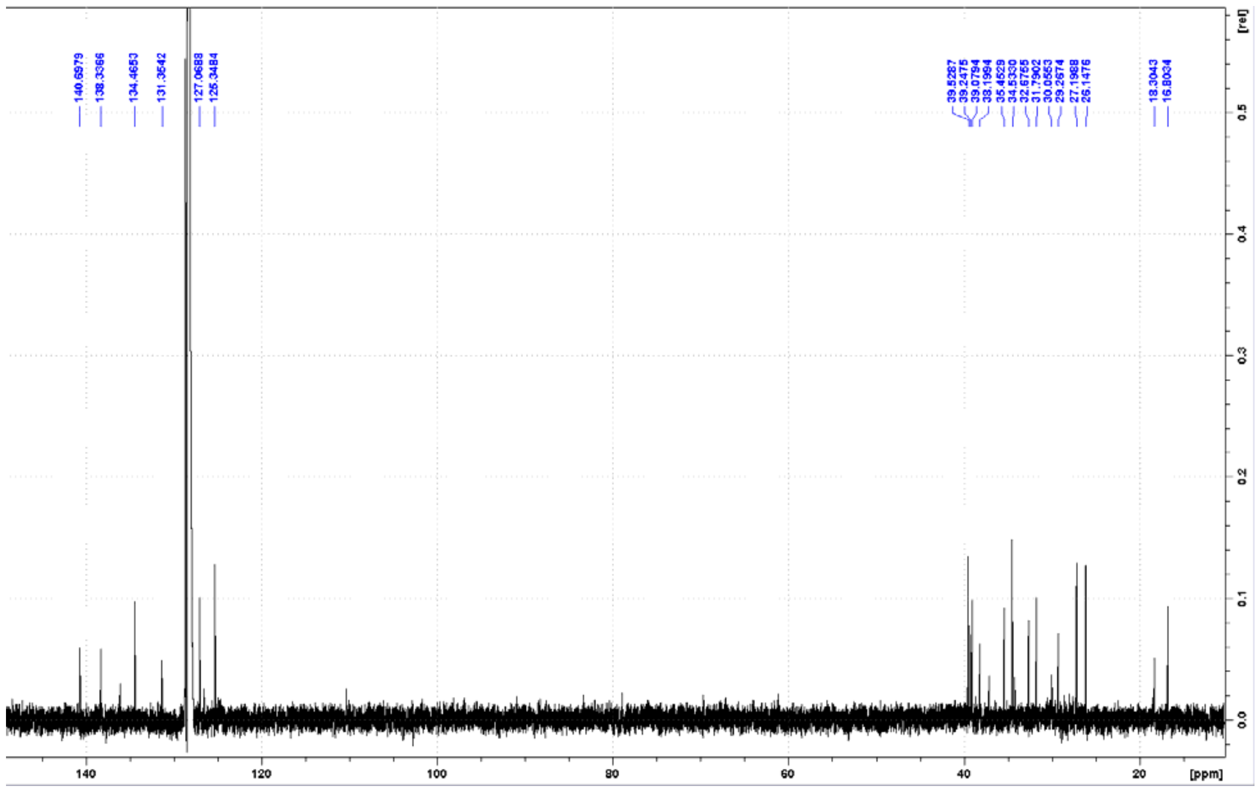

Figure S7. ^{13}C spectrum of the TPS20c major diterpene compound (solvent C_6D_6).

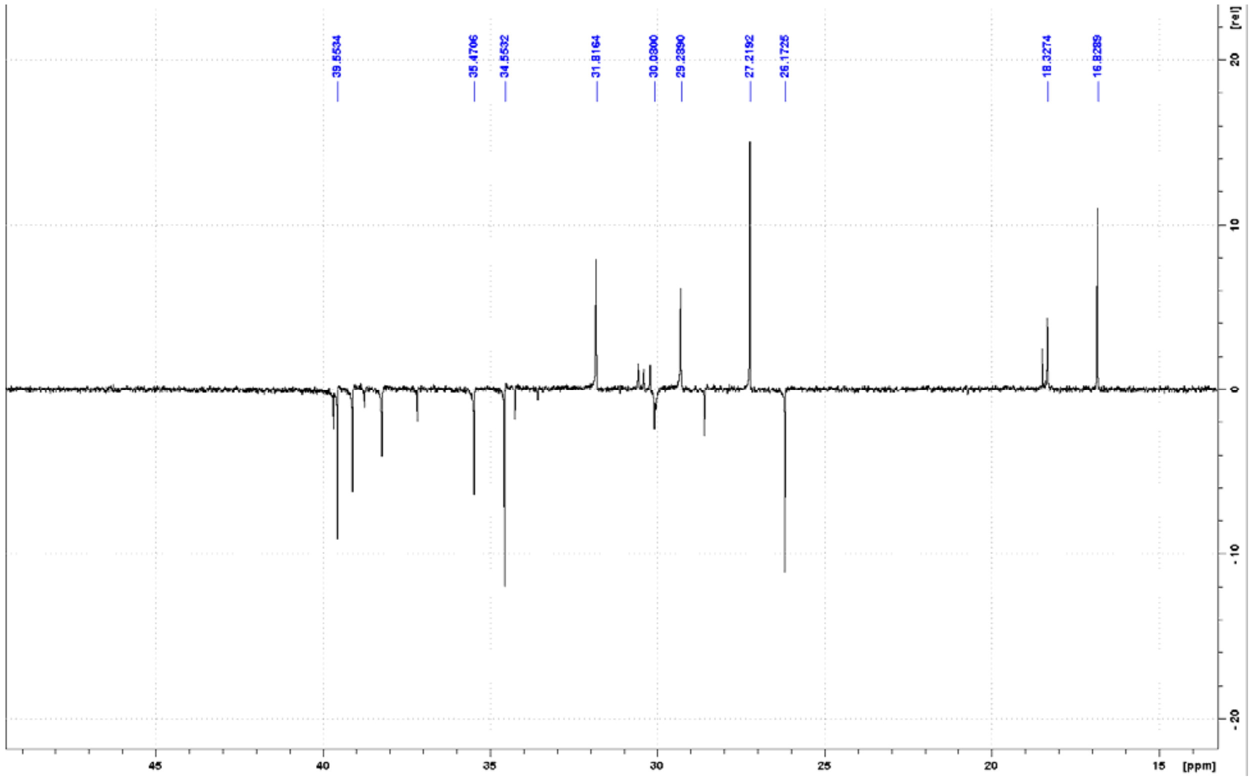

Figure S8A. ^{13}C DEPT135 of the TPS20c major diterpene compound (solvent C_6D_6).

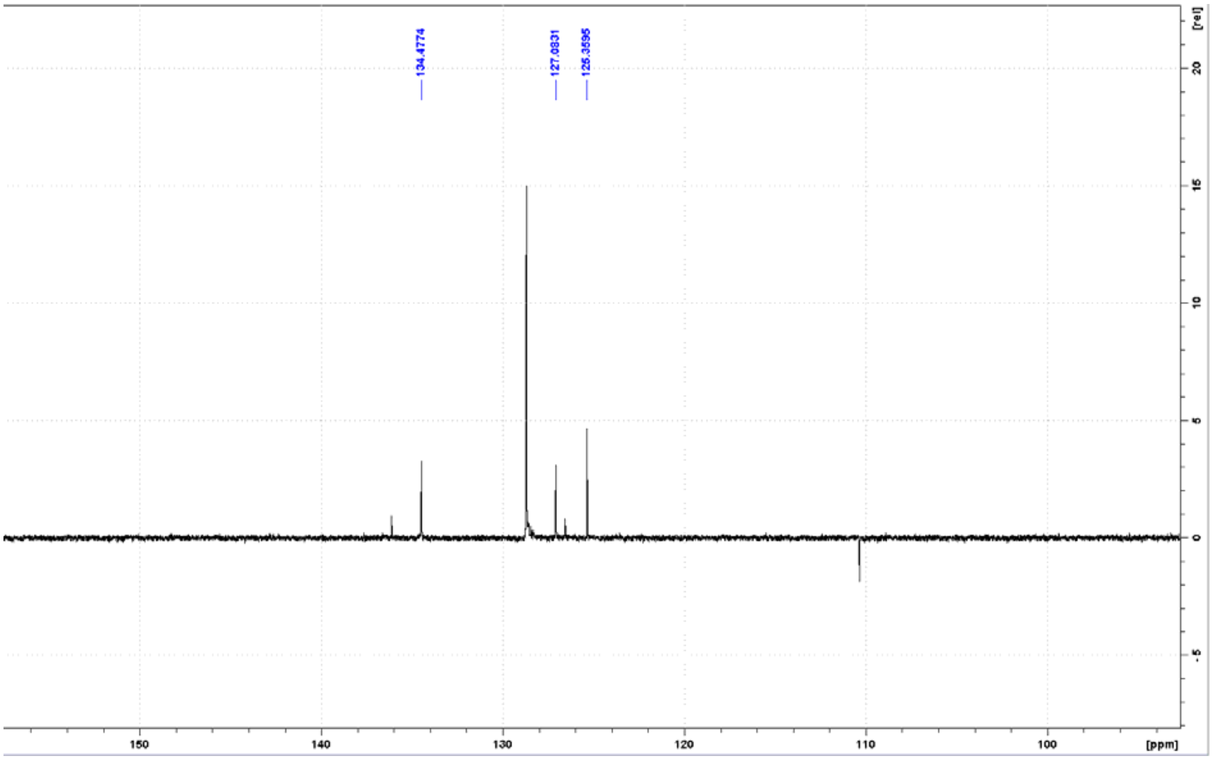

Figure S8B. ^{13}C DEPT135 of the TPS20c major diterpene compound (solvent C_6D_6).

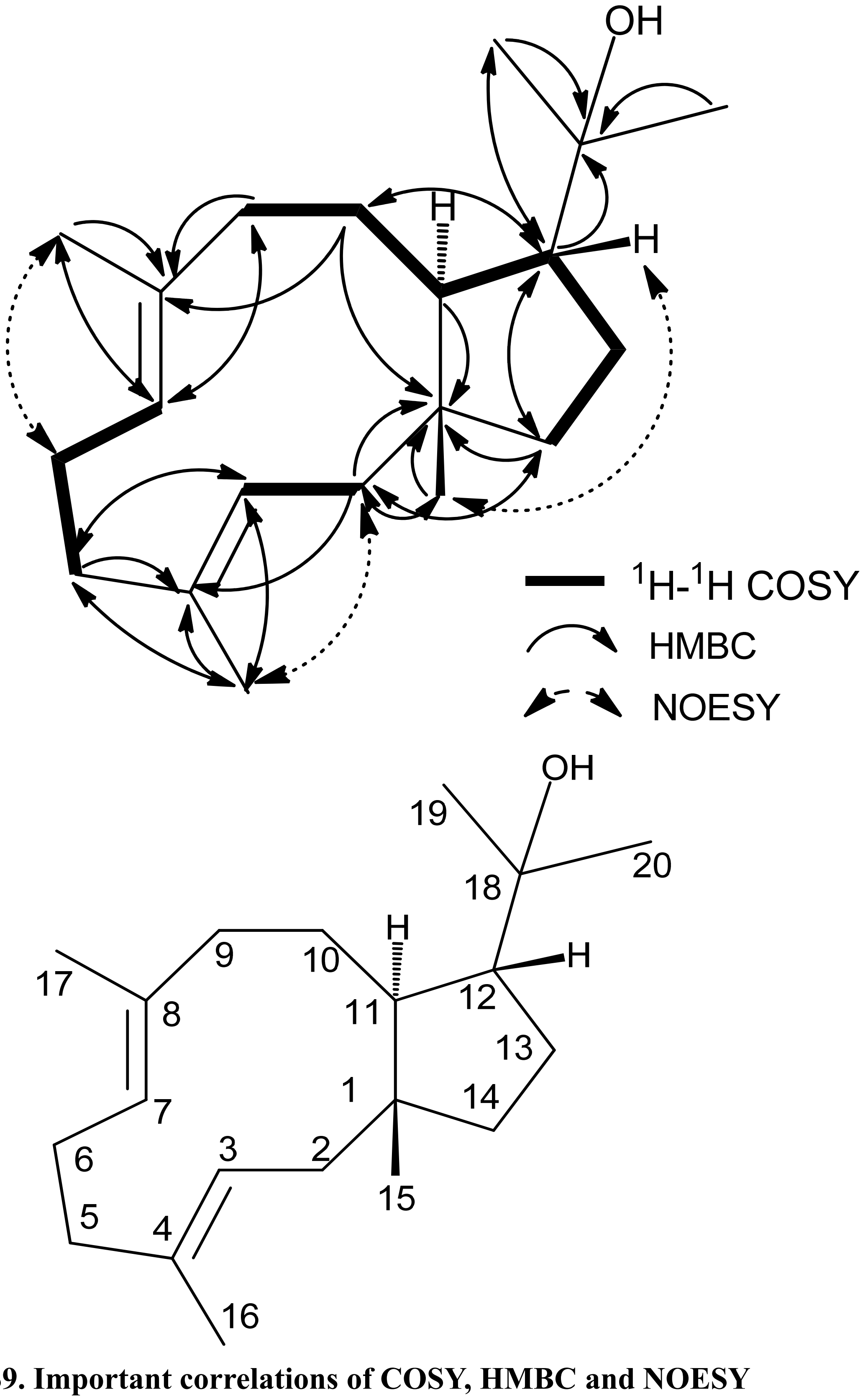

Figure S9. Important correlations of COSY, HMBC and NOESY observed for the major diterpene alcohol product of TPS20c.

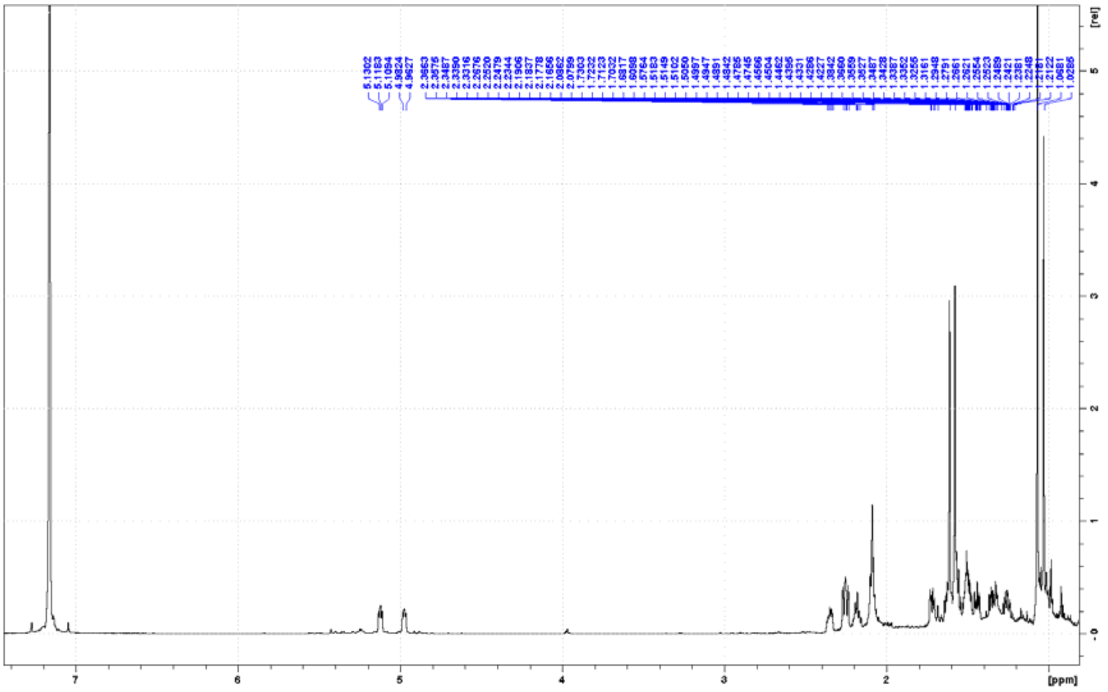

Figure S10. ^{1}H spectrum of the TPS20c diterpene alcohol compound (solvent C_6D_6).

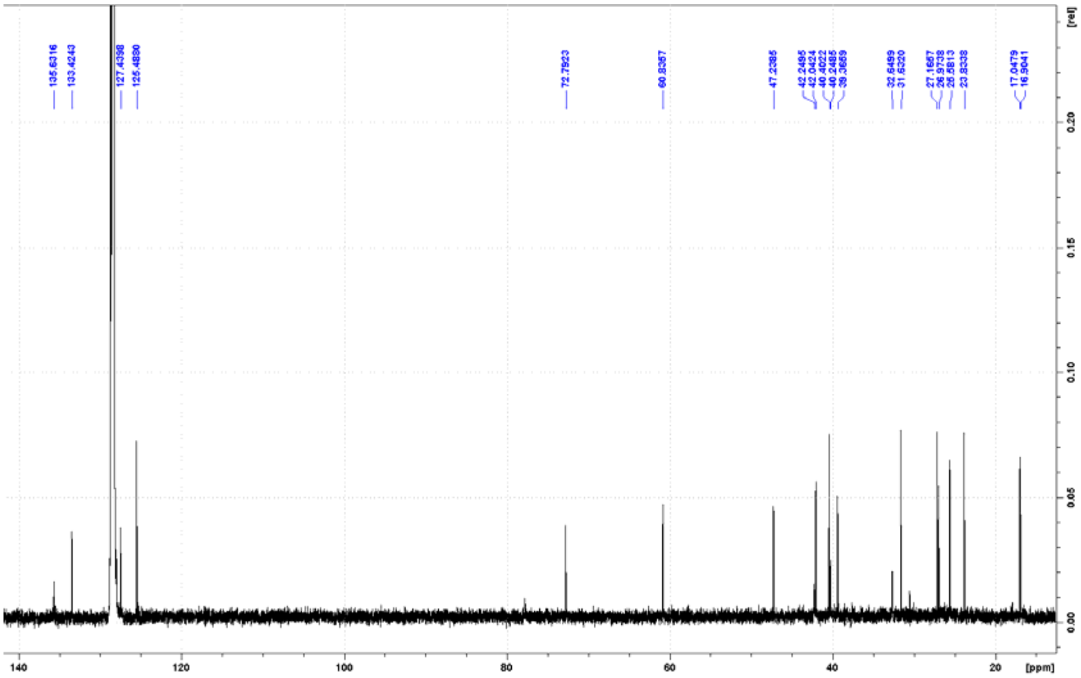

Figure S11. ^{13}**C spectrum of the TPS20c diterpene alcohol compound (solvent C_6D_6).**

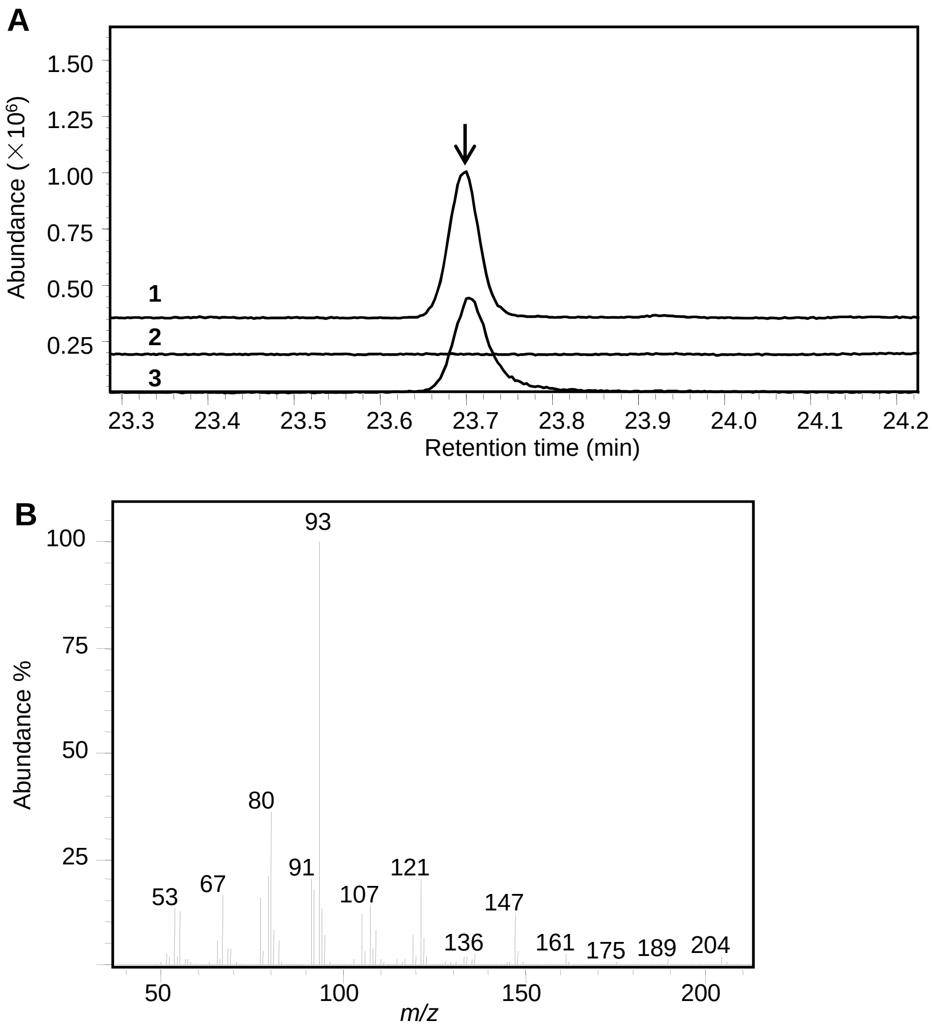

Figure S12. TPS20c reacts with (*E*,*E*)-FPP to produce α-humulene. (A) GC-MS chromatogram of an extract from *E. coli* coexpressing TPS20c and (*E*,*E*)-FPP synthase. 1, co-expression of TPS20c and (*E*,*E*)-FPP synthase. 2, co-expression of empty vector and (*E*,*E*)-FPP synthase. 3, standard of α-humulene. **(B)** Mass spectrum of α-humulene (arrow marked).

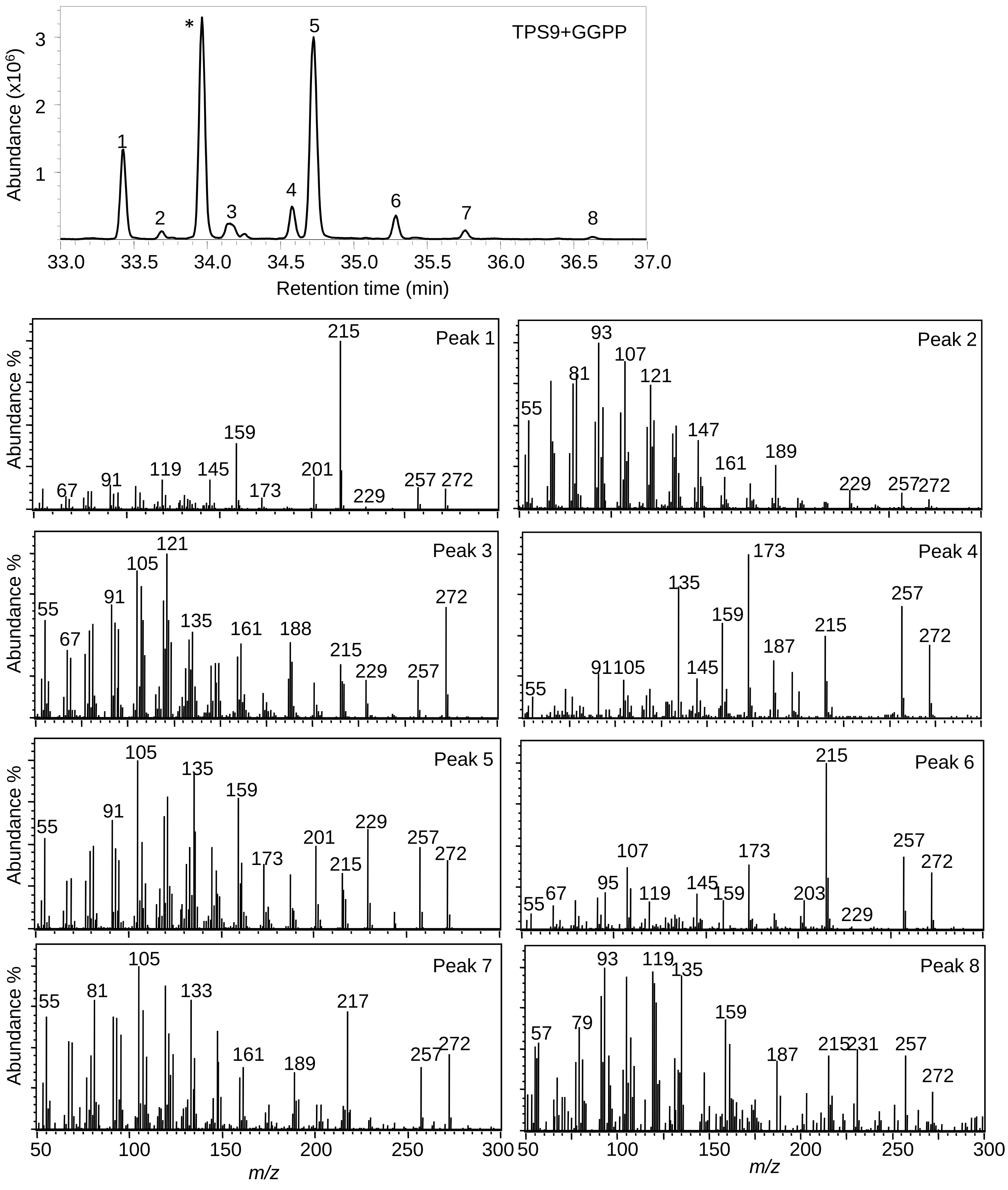

Figure S13. Mass spectra of minor diterpene products of recombinant TPS9.
The mass spectrum of the major diterpene product (labeled with asterisk) is shown in Figure 7C.

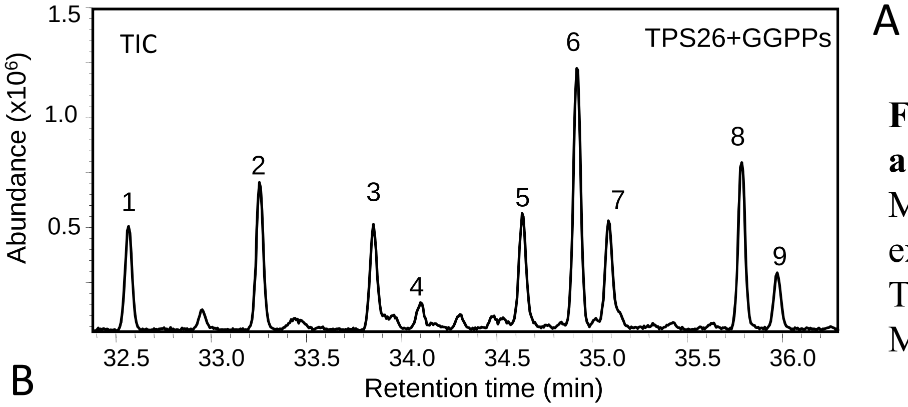

Figure S14. Diterpene synthase activity of Col TPS26. (A) GC-MS chromatogram of a culture extract from *E. coli* coexpressing TPS26 and GGPP synthase. (B) Mass spectra of TPS26 products.

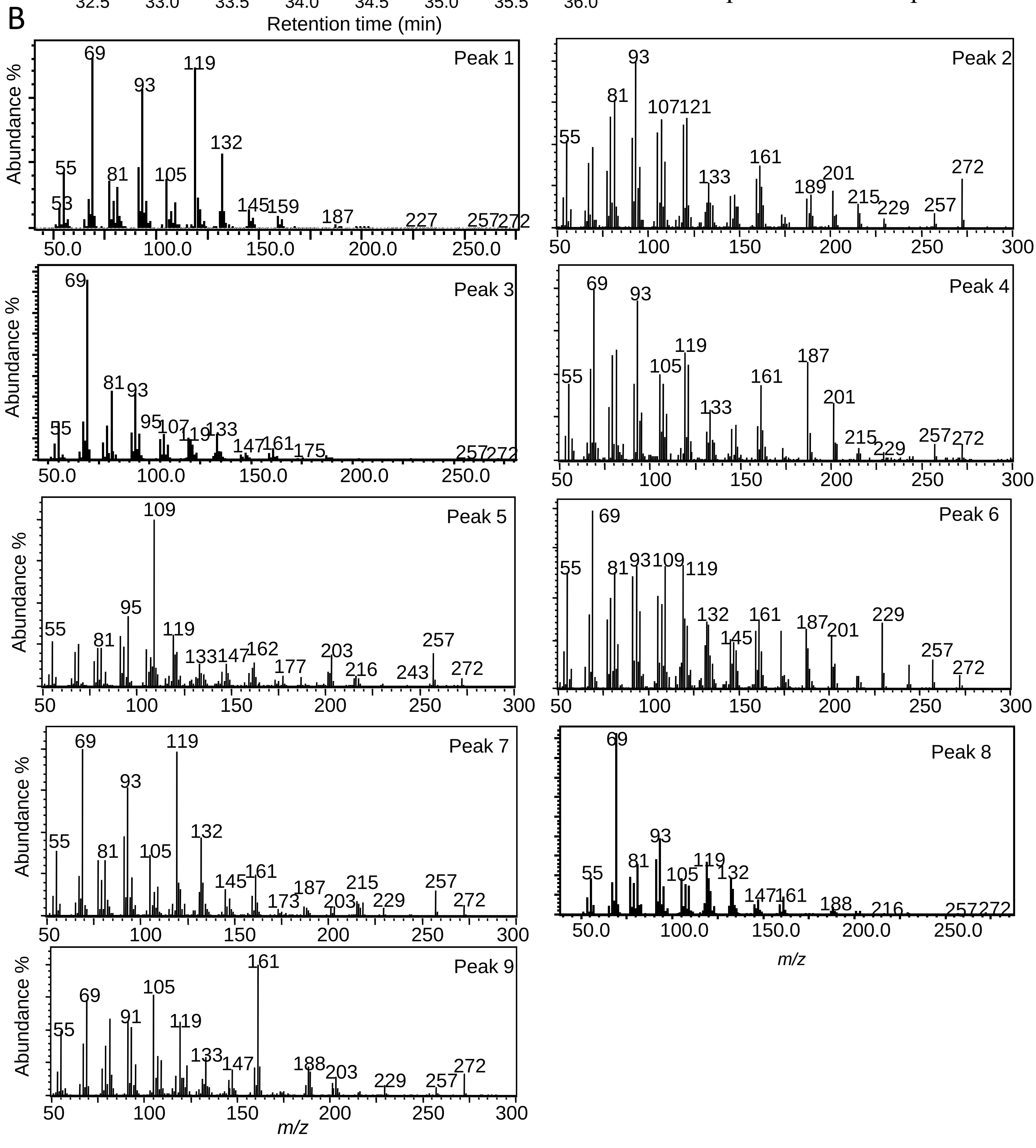

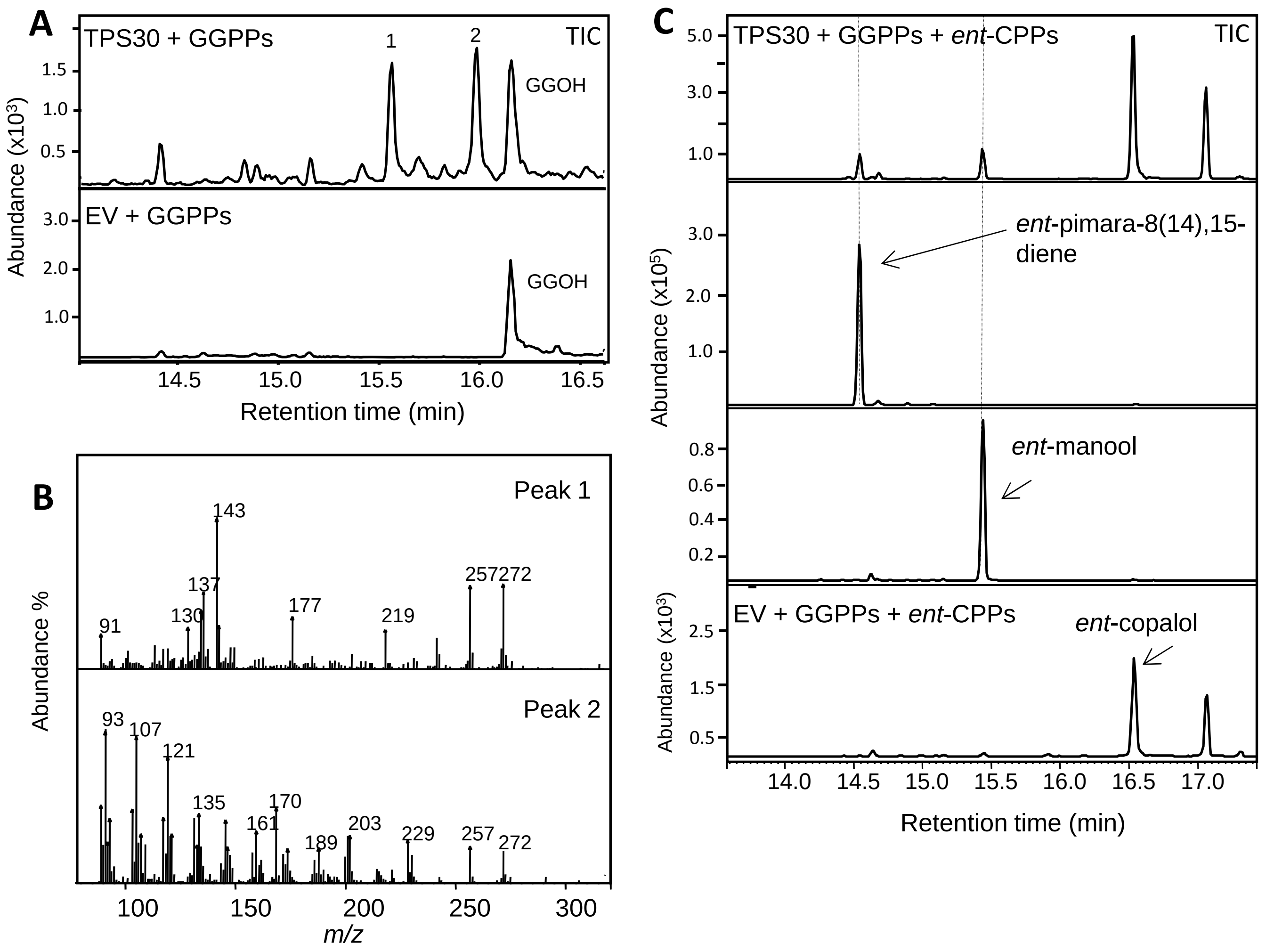

Figure S15. Diterpene synthase activity of Col TPS30. (A) Upper panel: GC-MS chromatogram of a culture extract from *E. coli* coexpressing truncated TPS30 and GGPP synthase. Lower panel: Extract of *E. coli* expressing only GGPP synthase. (B) Mass spectra of the TPS30 diterpene products 1 and 2 as shown in (A). (C) Upper panel: GC-MS chromatogram of a culture extract from *E. coli* coexpressing truncated TPS30, GGPP synthase and *ent*-CPP synthase. Diterpene products are marked with lines and arrows corresponding to authentic standards. Lower panel: Extract of *E. coli* expressing only GGPP synthase and *ent*-CPP synthase. EV, empty vector.

Table S1. Primers used in this study.

Primer name	Sequence 5'-3'
**RT-PCR**	
TPS6-F	GAGGTCGGGGTAGCTTCG
TPS6-R	CTCTCCAGCGAAGGTGTACC
TPS19-F	GCCACGTACCAACCTTTGATG
TPS19-R	GGGTATTGGGTGGACGTACAAG
TPS20-F	GTTGGAGGCAGCAATGAGGAAC
TPS20-R	CTAAATGGTGATTAGATCAAGG
TPS29-F	GTGTGAAACAAGTGGTAGAGAAG
TPS29-R	GGGTATAGGGTGGAGGTACAAG
Actin 8-F	ATGAAGATTAAGGTCGTGGCAC
Actin 8-R	GTTTTTATCCGAGTTTGAAGAGGC
**Cloning of full length and truncated *TPS* cDNAs**	
TPS6-F	AAAGTCGACATGGAGGCCATAACAAAATATG
TPS6-F-truncated	AACCATGGCAACGACGAATCCTACAGA
TPS6-R	AAACTCGAGTTAAAGAGTGATGAGATCGACA
TPS9-F-truncated	AACATATGGATGATAAAGAACGTACTCGC
TPS9-R	AACTCGAGCTACAACATTTTCAGAGTATGAT
TPS19-F	AACCATGGAAGCAACAAGAATGGGT
TPS19-F-truncated	AACCATGGCATCTACTAAGAGTAGTGATGATC
TPS19-R	AACTCGAGTTATGAAAGGGGTATTGGGTG
TPS20-F	AACCATGGAAGCAATAACTAAAAATGGG
TPS20-F-truncated	AACCATGGATCATGAAGCCGCAAATCGT
TPS20-F-truncated (His-tag)	AACATATGGATCATGAAGCCGCAAATCGT
TPS20-R	AAACTCGAGCTAAATGGTGATTAGATCAAGGA
TPS22-F-truncated	CACCACTCTTTCCCGGAGATCAA
TPS22-R	TCAAAGCGGAAGAGGATGGAAG
TPS25-F-truncated	CACCAACTTCTCTCTCTTTCCTTGC
TPS25-R	TCAAAGAGGTATTGGATGGAGG
TPS26-F-truncated	AACCATGGCACTTCGTTTAAAGGCCACTAATAC
TPS26-R	AAACTCGAG TCAAAGACCGATACGATCGA
TPS29-F	AACCATGGAAGCAATAAGAATAGGTTT
TPS29-F-truncated	AACCATGGCAAGTTCTTCTAAGAAGAGTA
TPS29-R	AACTCGAGTTATGAAAGGGGTATAGGGTG
TPS30-F	AACCATGGCAGTAGCAAGAACGGT
TPS30-F-truncated	AACCATGGCTACTACTAAGAGTTCGACT
TPS30-R	AACTCGAGTCAAACAGGAATGGGATGGAT

Table S2. ^{1}H-NMR and ^{13}C-NMR assignments for the major diterpene product of TPS20c (solvent: benzene-d_6).

position	δ_C (ppm)	δ_H (ppm)	Multiplicity, J in Hz
1	39.2	-	
2	39.1	2.01(2H)	m
3	127.1	4.84	t, 5.6
4	131.4	-	
5	39.5	1.93 (2H)	m
6	26.1	2.03	m
		2.07	m
7	125.3	4.91	t, 8.4
8	138.3	-	
9	38.2	2.33	m
		2.16	m
10	30.0	1.82	m
		2.02	m
11	140.7	-	
12	134.5	5.44	d, 1.24
13	32.7	-	
14	34.5	1.48 (2H)	m
15	35.4	1.33	m
		1.82	m
16	27.2	0.99(3H)	s
17	16.8	1.48(3H)	s
18	18.3	1.47(3H)	s
19	29.3	1.05(3H)	s
20	31.8	1.01(3H)	s

Table S3. ^{1}H-NMR and ^{13}C-NMR assignments for the major diterpene alcohol product of TPS20c (solvent: benzene-d_6).

position	δ_C (ppm)	δ_H (ppm)	Multiplicity, J in Hz
1	47.2	-	
2	40.2	1.72	dd, 13.5, 6.1
		2.25	dd, 13.5, 9.8
3	125.5	5.12	dd, 9.8, 6.1
4	133.4	-	
5	40.4	2.08 (2H)	m
6	25.6	2.09	m
		2.18	m
7	127.4	4.97	dd, 9.2, 5.6
8	135.6	-	
9	39.4	2.25	m
		2.35	m
10	32.6	1.49	m
		1.58	m
11	42.0	1.63	m
12	60.8	1.56	m
13	27.2	1.25	m
		1.50	m
14	42.2	1.35	m
		1.44	m
15	23.8	1.03(3H)	s
16	16.9	1.58(3H)	s
17	17.0	1.61(3H)	s
18	72.8	-	
19	27.0	1.07(3H)	s
20	31.6	1.07(3H)	s
